# Bidentate Donor-Functionalized *N*-Heterocyclic Carbenes: Valuable Ligands for Ruthenium-Catalyzed Transfer Hydrogenation

**DOI:** 10.3390/molecules27154703

**Published:** 2022-07-23

**Authors:** Vincent Ritleng, Christophe Michon

**Affiliations:** Ecole Européenne de Chimie, Polymères et Matériaux, Université de Strasbourg, CNRS, LIMA, UMR 7042, 25 Rue Becquerel, 67087 Strasbourg, France; cmichon@unistra.fr

**Keywords:** ruthenium, bidentate *N*-heterocyclic carbene, transfer hydrogenation

## Abstract

Ruthenium complexes are by far the most studied compounds that catalyze hydrogen transfer reactions. In this review, we describe the use in this field of ruthenium complexes bearing bidentate donor-functionalized *N*-heterocyclic carbene ligands. The review specifically covers the application in transfer hydrogenations of (*κ*^2^-*C_NHC_*,*Y*)-ruthenacyclic compounds where the Y donor atom is a N, P, O, or S atom, and where the *N*-heterocyclic carbene ligand is a classical imidazol-2-ylidene, a benzimidazol-2-ylidene, a mesoionic 1,2,3-triazolylidene, or an imidazol-4-ylidene ligand. Tridentate donor-functionalized *N*-heterocyclic carbene complexes thus fall outside the scope of the review. Applications in (asymmetric) transfer hydrogenation of ketones, aldehydes, imines, alkenes, and nitrobenzene are discussed.

## 1. Introduction

Transition-metal catalyzed transfer hydrogenation (TH) is a key reaction for the fine chemical and pharmaceutical industries as a safer alternative to hydrogenation of multiple bonds with flammable hydrogen gas [1]. The TH of aldehydes, ketones, and imines in particular are industrially relevant. These reactions are usually carried out with isopropanol as both the solvent and the hydrogen source. Other common hydrogen donors include formic acid or, more recently, glycerol. Ruthenium is undoubtedly one of the most exploited transition metals, together with iridium and rhodium, for this transformation [2,3,4,5,6,7], and a wide variety of ligands have been used with it [3,5,6,7]. Among them, *N*-heterocyclic carbenes are interesting for affording high thermal stability and tunability of the steric and electronic properties of the metal [6]. Nevertheless, several common decomposition pathways exist for metal–NHC complexes [8], which limits the ability of monodentate NHC ligands to effectively stabilize the ruthenium centers at the high temperatures often employed in TH reactions, i.e., in refluxing 2-propanol or formic acid. In this context, the ability of bidentate donor-functionalized NHCs to stabilize metal centers by preventing decomposition and offering the possibility to act as a hemilabile ligand with reversible dissociation of the non-carbon atom is quite valuable [9,10] and has been largely exploited in ruthenium-catalyzed TH reactions. This review provides a comprehensive overview of the advances in this field with (*κ*^2^-*C_NHC_*,*Y*)-ruthenacyclic precatalysts where the Y donor atom is a N, P, O, or S atom, and where the *N*-heterocyclic carbene ligand comprises (benz)imidazol-2-ylidenes, mesoionic 1,2,3-triazolylidenes, and imidazol-4-ylidenes.

## 2. Transfer Hydrogenation of Ketones

As it will appear to the reader, the large majority of the literature for this reaction deals with cationic half-sandwich complexes that bear chelating *N*-functionalized imidazole-2-ylidene or mesoionic triazolylidene ligands.

### 2.1. Ketones’ Transfer Hydrogenations with (κ^2^-C_NHC_,N)-Complexes

**Scheme 1:**
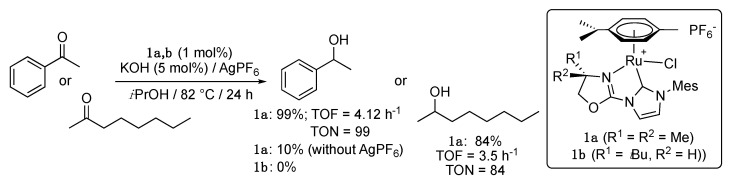
TH of acetophenone and 2-octanone catalyzed by the oxazolinyl-functionalized complex **1a**.

The first example of this long series was reported in 2006 by Gade et al. with the description of the synthesis—by transmetalation from the corresponding silver NHC complexes—and catalytic activity of the *p*-cymene-ruthenium complexes **1a**,**b** bearing an oxazolinyl-functionalized imidazole-2-ylidene ligand [11]. Complex **1a** showed moderate activity in the TH of acetophenone and 2-octanone from *i*PrOH/KOH at 82 °C in the presence of AgPF_6_, achieving turnover numbers (TON; expressed as the yield or conversion (%) after a given time/catalyst loading (mol%)) from 84 to 99, and turnover frequencies (TOF; expressed as the TON/reaction time (h)) from 3.5 to 4.12 h^−1^ (Scheme 1). However, the reaction failed with the more sterically demanding pinacolone. Interestingly, the formation of a dicationic species seemed to be crucial, since the use of **1a** in the absence of AgPF_6_ led to very poor yields. On the other hand, the chiral complex **1b** bearing a bulky *t*-butyl group proved completely unreactive under these conditions.

**Scheme 2:**
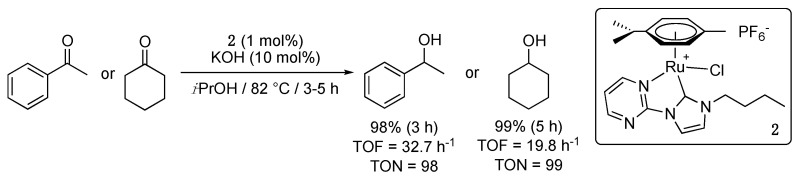
TH of acetophenone and cyclohexanone catalyzed by the pyrimidine-functionalized complex **2**.

The second example only appeared three years later with a contribution of Crabtree et al. that disclosed the pyrimidine-functionalized imidazol-2-ylidene-Ru(II) complex **2** [12]. This air-stable complex, which was similarly prepared by transmetalation from the corresponding silver carbene complex, was found to be relatively more active than **1a** achieving almost complete reduction in acetophenone and cyclohexanone in only 3 to 5 h in 2-propanol at 82 °C. However, it is noteworthy that 10 mol% of KOH as base were required (Scheme 2).

**Scheme 3:**
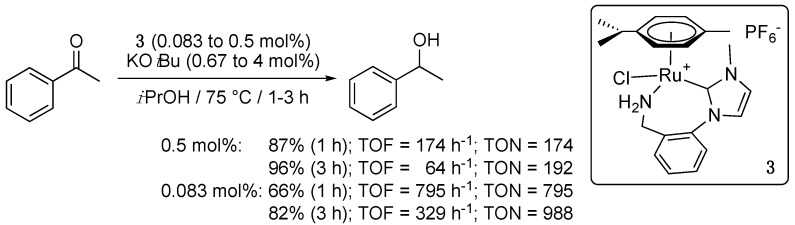
TH of acetophenone by the amino-functionalized NHC-Ru(II) complex **3**.

At about the same period, Morris et al. reported the first paper of a long series dealing with the use of half-sandwich Ru complexes bearing primary amino-functionalized NHC ligands in both TH and H_2_-hydrogenation catalysis [13]. By transmetalation of the amino-functionalized NHC ligand from the corresponding homoleptic bis-NHC-nickel(II) complex to [Ru(η^6^-*p*-cymene)Cl_2_]_2_, they prepared the cationic air-stable Ru(II) complex **3** in 60% yield. The latter proved inactive at room temperature, but efficiently catalyzed the TH of acetophenone at 75 °C in the presence KO*t*Bu and 2-propanol, allowing 87% conversion in 1 h with a catalyst loading of 0.5 mol% (TOF = 174 h^−1^), and 66% in 1 h with a loading of only 0.083 mol% (TOF = 795 h^−1^) (Scheme 3). Notably, the catalytic activity of **3** was barely affected by the nature of the strong base used (KO*t*Bu, NaO*i*Pr, or KOH), or the presence of small amounts of water, which were generated when KOH was used as a base.

A couple of years later the same group disclosed another cationic amino-functionalized imidazol-2-ylidene-Ru(II) complex **4a**, in which the chelating carbene ligand formed a six-membered ring instead of a seven-membered ring in complex **3** [14]. This complex, which was also synthesized by transmetalation from the adequate silver carbene species, showed a slightly lower activity than **3** under the same conditions (0.5 mol% **4a**, 4 mol% KO*t*Bu, 75 °C, in 2-propanol), with 49% conversion of acetophenone to 1-phenylethanol in 1 h (TOF = 98 h^−1^) and 92% conversion in 3 h (TOF = 61.3 h^−1^). A more comparable activity was observed with a catalyst loading of 0.083 mol%, with 53% conversion after 1 h (TOF = 636 h^−1^) and 90% after 5 h (TOF = 216 h^−1^).

**Scheme 4:**
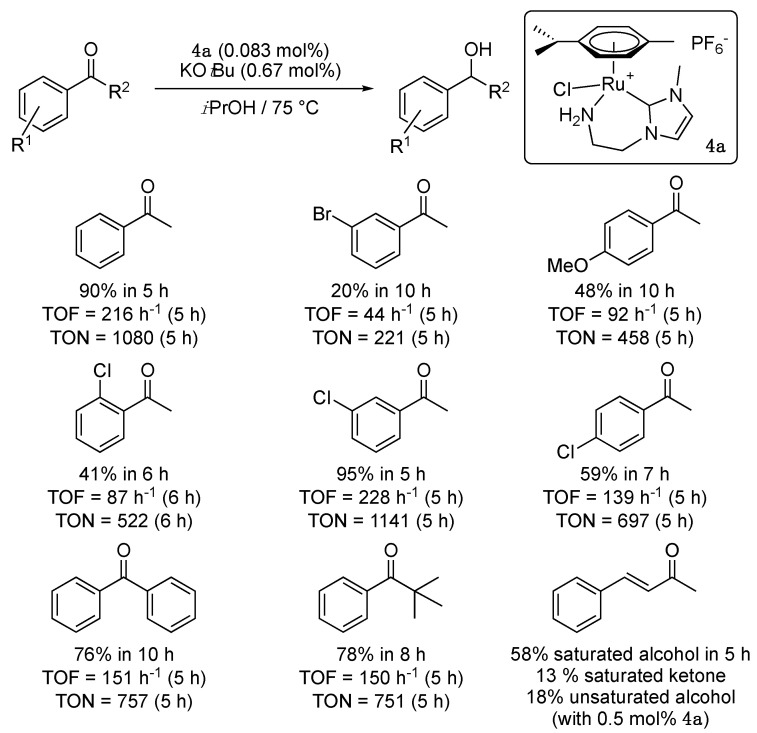
TH of ketones by the amino-functionalized imidazol-2-ylidene complex **4a**.

A brief study of the reaction scope, carried out with a catalyst loading of 0.083 mol%, showed that TH was difficult for acetophenones that contained an electron-donating 4′-methoxy or a reactive bromide group on the phenyl group (Scheme 4). The 2′- and 4′-chloroacetophenones proved difficult to hydrogenate compared to 3′-chloroacetophenone. Good conversions could be achieved with benzophenone and 2,2-dimethylpropiophenone, yet after long reaction times. Finally, the reduction in *trans*-4-phenylbut-3-ene-2-one was unselective, giving predominantly the saturated alcohol, 4-phenylbutan-2-ol, which is characteristic of an inner-sphere mechanism involving the decoordination of the amine side arm.

The corresponding hydride complex **4b** was prepared by reaction of **4a** with 3 equiv. of NaO*i*Pr in 2-propanol at 50 °C. Interestingly, it showed little reactivity in the absence of a base, giving only 22% conversion of acetophenone to 1-phenylethanol in 4 h in the presence of a 0.5 mol% catalyst loading, and about 10% conversion under stoichiometric conditions in THF-*d*_8_ at 50 °C. This low reactivity, together with the large free energy barrier (ΔG^≠^) that was computed by DFT for a H^+^/H^−^ couple to transfer to acetophenone from a model amine-hydride complex militated against an outer-sphere bifunctional mechanism. In contrast, the favorable energy barriers computed for the two crucial steps of an inner-sphere mechanism involving the decoordination of the amine side arm, namely (i) the β-hydride elimination of a coordinated 2-propoxide ligand, and (ii) an attack of the resulting hydride on the coordinated acetophenone, strongly militated in favor of the latter [14].

**Scheme 5:**
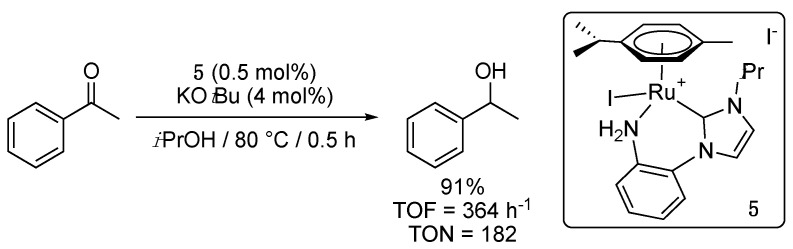
TH of acetophenone catalyzed by the amino-functionalized NHC-Ru(II) complex **5**.

During the same period, Cross and his collaborators communicated about the related aniline-functionalized NHC-Ru(II) complex **5** [15], displaying also a six-membered metalacyclic ring. The latter demonstrated higher catalytic activity than **3** and **4a** with 93% conversion of acetophenone to 1-phenylethanol in half an hour only, under similar conditions, i.e., 0.5 mol% **5**, 4 mol% KO*t*Bu, 80 °C, in 2-propanol (Scheme 5). Furthermore, **5** proved much more active than the corresponding Cp* (Cp* = η^5^-C_5_Me_5_) Rh complex, and the related Cp*Ir-amide complex. Similarly to **3** and **4a**, **5** however showed no activity at room temperature.

**Scheme 6:**
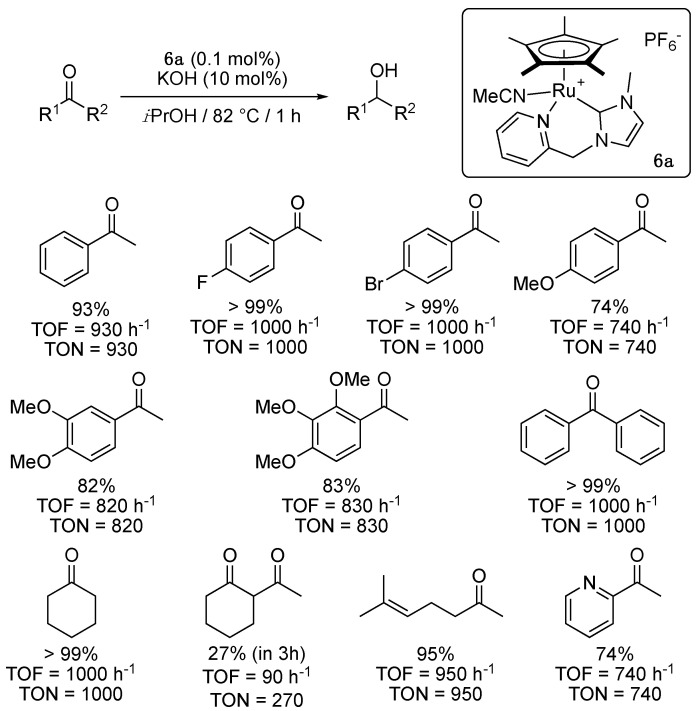
TH of ketones catalyzed by the picolyl-functionalized NHC-Ru(II) complex **6a**.

In 2011, Valerga and Puerta reported the synthesis and characterization of a series of picolyl-functionalized NHC-Ru(II) complexes **6** bearing a Cp* (η^5^-C_5_R_5_) ligand [16]. The cationic complexes were synthesized by treatment of [RuCp*(CH_3_CN)_3_][PF_6_] with a THF solution of the appropriate ligand, in situ generated by the reaction of the picolylimidazolium bromide and an excess of KO*t*Bu. All tested picolylcarbene complexes were shown to catalyze the TH of acetophenone from isopropanol at 82 °C with KOH as base. Notably, the complex **6a** containing a methyl group as a wingtip substituent and hydrogen atoms on the imidazole backbone was found to be more active than those containing an isopropyl (**6b**) or mesityl group (**6c**) as a wingtip substituent and those containing chloro substituents on the backbone and a methyl group as a wingtip substituent (**6d**), with a turnover frequency value at 50% conversion (TOF_50_) of 62 min^−1^ vs. TOF_50_ of 0.38 to 20 min*^−^*^1^ for **6b**–**d** with a catalyst loading of 1 mol%. This showed that both steric and electronic have a non-negligible influence on the catalytic activity of these family of complexes. A maximum TOF_50_ of 100 min^−1^, i.e., 6000 h^−1^, could even be observed for the TH of acetophenone with a catalyst loading of 0.1 mol%, and **6a** was able to reduce a variety of aromatic and aliphatic ketones with a remarkable efficacy compared to the previously described complexes **1**–**5** (Scheme 6).

Regarding the reaction mechanism, no experimental data was acquired [16], but some insight to the TH of acetophenone catalyzed by **6a** was provided by a DFT study at the B3LYP level of theory conducted by Shang and Xu [17]. From the results of the calculations, it was concluded that a stepwise inner-sphere monohydride mechanism involving decoordination of the pycolyl arm as an entry point should be favored from an energetic point of view.

**Figure 1:**
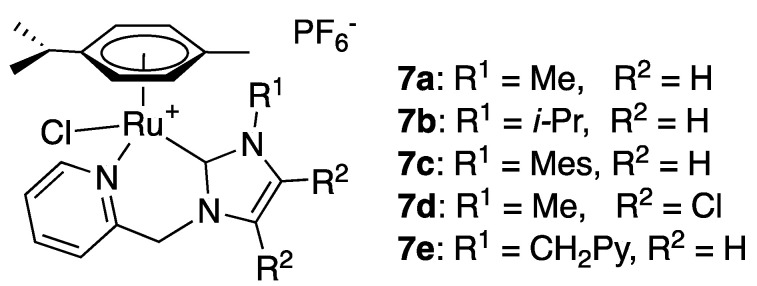
(η^6^-*p*-cymene)Ru(II) complexes **7a**–**e** bearing picolyl-NHC ligands.

A year after their first study, Puerta and Valerga published a second article dealing with picolyl-imidazol-2-ylidene complexes, but this time with a η^6^-*p*-cymene instead of a Cp* as the capping ligand [18]. The results were in the same vein as those obtained with complexes **6a**–**d** regarding the effect of the wingtip and imidazole backbone substituents on the comparative activity of complexes **7a**–**d** (Figure 1) and the reaction scope. The activity was slightly lower though, as the most active complex **7a** required a catalyst loading of 0.2 mol% and reaction times of 6–24 h to reach similar yields as those obtained with **5a** in 1 h with a catalytic loading of only 0.1 mol% (Scheme 6). The more recently reported complex **7e** bearing a second (uncoordinated) picolyl side arm as wingtip substituent proved, in contrast, to be almost unreactive, as only a 15% reduction in acetophenone was achieved after 16 h reaction with a catalyst loading of 1 mol% in the presence of KO*t*Bu (5 mol%) in isopropanol at 80 °C [19].

**Scheme 7:**
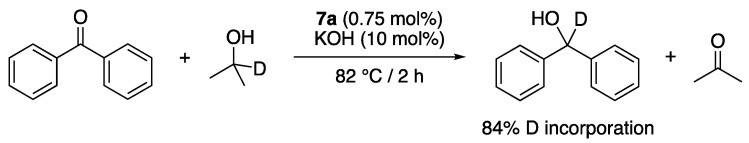
Deuterium labeling experiment with **7a** establishing a monohydride mechanism.

The reasons for the lower activity of complexes **7a**–**d** compared to their Cp* analogues **6a**–**d** were not discussed. However, the application of the deuterium labeling protocol—first described by Bäckvall [20] and Crabtree [21]—to distinguish between possible monohydride and dihydride reaction mechanisms, allowed the authors to establish a monohydride mechanism with **7a** [18], in line with the results of the DFT calculations conducted by Shang and Xu with **6a** [17]. Indeed, when a monohydride mechanism is operating, the C–H bond of the hydrogen donor should end up exclusively as the C–H bond on the carbinol carbon of the product, whereas when a dihydride mechanism is operating, the donor C–H bond should be scrambled between the carbinol C–H and the O–H bonds, and the TH of benzophenone from 2-propanol-2-*d* in the presence of **7a** resulted in the formation of mostly monodeuterated 1,1-diphenylmethanol-1-*d* with 84% deuterium incorporation at the carbinol position (Scheme 7) [18].

**Scheme 8:**
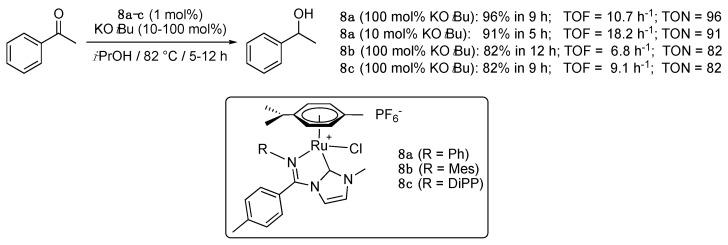
TH of acetophenone catalyzed by the imino-functionalized imidazol-2-ylidene-Ru(II) complexes **8a**–**c**.

The synthesis, via transmetalation from the corresponding silver complexes, and characterization of a series of three five-membered imino-functionalized imidazol-2-ylidene-Ru(II) complexes **8a**–**c** bearing a η^6^-*p*-cymene ligand, and of the corresponding Cp* iridacycles was reported by Hou et al. in 2012 [22]. All complexes showed relatively modest activity (>80% in 9–12 h) for the TH of acetophenone from isopropanol at reflux with a catalyst loading of 1 mol% in the presence of 1 equiv. of KO*t*Bu as base. As observed with complexes **6a**–**d** and **7a**–**d**, the complex **8a** with the smallest steric footprint proved to be the most effective with a yield of up to 96% in 9 h under these conditions, and of up to 91% in 5 h in the presence of 10 mol% KO*t*Bu (Scheme 8). Under these optimized conditions (10 mol% KO*t*Bu), **8a** reduced a small range of seven aromatic ketones, as well as the aliphatic ketone, 2-decanone, with good to excellent yields. No mechanistic details were given.

**Scheme 9:**
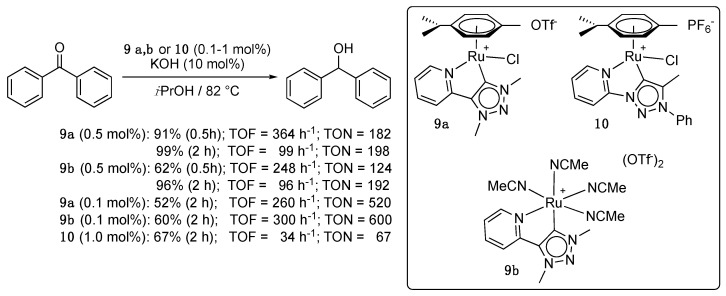
TH of benzophenone catalyzed by the pyridyl-functionalized mesoionic triazolylidene complexes **9a**,**b** and **10**.

The first examples of donor-functionalized mesoionic triazolylidene ruthenium precatalysts for ketone TH appeared in 2014 [23]. As a subclass of NHCs, mesoionic 1,2,3-triazolylidene ligands generally present slightly stronger σ-donor properties compared to classical imidazolium-derived systems and are easily accessed by click chemistry. In this early report, two pyridyl-functionalized triazolylidene (η*^6^*-*p*-cymene)Ru complexes **9a** and **10**, that differ by the bonding of pyridyl unit to either the triazole nitrogen or carbon, were synthesized by transmetalation from the corresponding silver carbenes and shown by Albrecht et al. to catalyze the TH of benzophenone with low to good efficiency depending on their structure (Scheme 9). Hence, the C-bonded pyridyl complex **9a** achieved a TON as high as 520 in 2 h in refluxing isopropanol whereas the N-bonded derivative **10** gave at most a TON of 67 in the same amount of time. The octahedral derivative of **9a**, the *p*-cymene-free complex **9b** bearing four acetonitrile ligands, showed a comparable activity to this latter. The relatively high activity of **9a**/**b** was attributed to the relatively low electron density of the ruthenium center, which would facilitate the isopropoxide anion coordination. Time-dependent monitoring of the reaction conducted with a **9a** loading of 0.5 mol% revealed a TOF_50_ of 680 h^−1^, a value that is lower by a 10-fold order to that observed with the picolyl-NHC complex **6a** [16], and also lower to the values reported for the amino-NHC complexes **3** [13] and **4a** [14]. No induction period was revealed by these kinetic measurements, and pseudo-first order kinetics was observed for both substrate consumption and product formation. A short reaction scope study demonstrated that diaryl, aryl-alkyl, and dialkyl ketones (7 examples) are readily hydrogenated under these conditions [23].

**Scheme 10:**
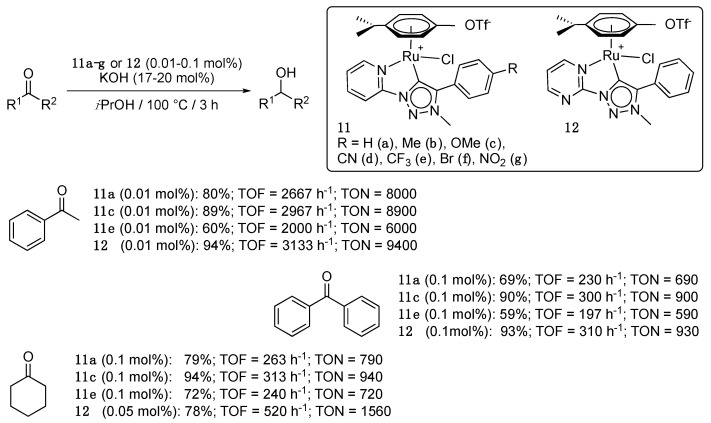
TH of various ketones catalyzed by the pyridyl- or pyrimidyl-functionalized mesoionic triazolylidene complexes **11a**–**g** and **12**.

Shortly after Albrecht’s initial report, Sarkar et al. described a series of eight analogues of **10** with varying electronic properties, that showed greatly improved catalytic properties compared to **9a**,**b** and **10**, though under harsher conditions (100 °C, KOH (17–20 mol%)) [24,25].The complex **11c** (0.01 mol%) bearing an electron-donating methoxy substituent [24] and the pyrimidine derivative **12** [25], in particular, gave outstanding results with TON as high as 8900 and 9400, respectively, for the TH of acetophenone, while complexes **11a** and **11e** displayed slightly lower activities (Scheme 10), and complexes **11b**,**d**,**f**,**g** required a loading of 0.5 mol% to achieve high yields. The same trend was observed with the more sterically hindered benzophenone, with TON of 900 and 930, and with the aliphatic cyclohexanone, with TON of 940 and 1560, respectively, (Scheme 10). Notably, the catalytic activity of Cp*-iridium and osmium analogues of **11a**–**g** and **12** was also studied, and ruthenium worked better in all cases. Similarly to what was observed with the ruthenium complexes **11a**–**g**, osmium complexes bearing electron-donating substituents were found to be more active than those bearing electron-withdrawing substituents. For the iridium complexes, the electronic effects were reversed. The authors suggested that this trend could be related to the difference between the *p*-cymene and Cp* ligands in both systems. Finally, attempts to detect a Ru-hydride species by ^1^H NMR spectroscopy after the reaction of **11a** in the absence of substrate, but under otherwise similar conditions, failed. In contrast, the same procedure with the Os and Cp*-Ir species allowed detecting two hydride species in each case, therefore suggesting the intermediacy of such species in the reaction mechanism [24].

**Scheme 11:**
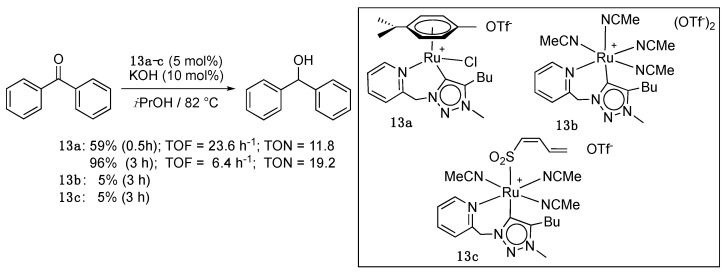
TH of benzophenone catalyzed by the picolyl-triazolylidene complexes **13a**–**c**.

The closely related picolyl-functionalized triazolylidene (η*^6^*-*p*-cymene)Ru complex **13a** (5 mol%), which was prepared via the usual transmetalation procedure from the adequate silver carbene, showed a much lower activity in comparison to complexes **9**–**12** with a maximum TON of 19.2 after 3 h reaction for the TH of benzophenone in the presence of 10 mol% KOH in refluxing isopropanol (Scheme 11) [26]. In addition, in contrast to **9b** vs. **9a**, the octahedral derivatives **13b** and **13c** bearing, respectively, four acetonitrile ligands and three acetonitrile and one butadienesulfonyl ligand showed almost no activity.

**Figure 2:**
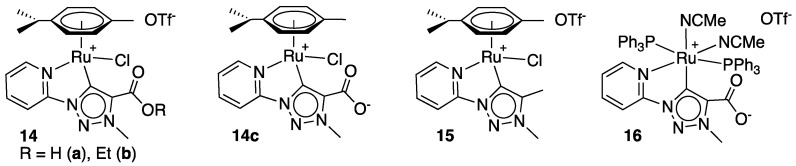
Pyridyl-functionalized triazolylidene complexes **14a**–**c** and **16** bearing a pendant carboxylic group and unfunctionalized complex **15**.

Having in mind the idea of bringing a non-coordinating Lewis base in close proximity to a Lewis acidic metal center in order to possibly access metal–ligand synergies, in particular for reversible substrate binding and proton release, Albrecht et al. introduced carboxylate groups to a pyridyl-triazolylidene ligand, closely resembling those found in complexes **10**–**12** [27]. The resulting complexes **14a**–**c** bearing an ester, carboxylic acid, or carboxylate functionality (Figure 2), were fully characterized and tested for the TH of acetophenone from isopropanol at reflux in the presence of KOH (10 mol%). The observed activity, although modest (95 to 97% conversion in 1 h with a catalytic loading of 1 mol%), suggested that the presence of a pendant carboxylic acid (**14a**) or ester group (**14b**) is beneficial for enhancing the catalyst activity when compared to that of the complex **15** (1 mol%) that does not bear a coordinating functionality (29% conversion in 1 h). The carboxylate derivative **14c** proved slightly less effective (75% conversion in 1 h), probably because this complex undergoes faster decarboxylation under basic conditions in comparison to **14a** and **14b**. Notably, the TH of acetophenone under base-free conditions with **14a** or **14c** led only to 0 and 9% conversion after 1 h, respectively, indicating that the pendant carboxylic group is not sufficiently basic for the formation of the reactive isopropoxide. The octahedral derivative **16** of the carboxylate complex **14c**, containing two labile acetonitrile and two triphenylphosphine ligands, displayed very low activity compared to the latter with only 16% conversion after 1 h. Furthermore, a TH experiment run with benzophenone in heptadeuterated isopropanol (CD_3_)_2_CDOH resulted in the exclusive and almost complete deuteration of the reaction product at the carbinol carbon, thus suggesting a monohydride mechanism [20]. Attempts to detect a ruthenium hydride species by reaction of **14b** in isopropanol in the presence of KO*t*Bu were however unsuccessful.

**Figure 3:**
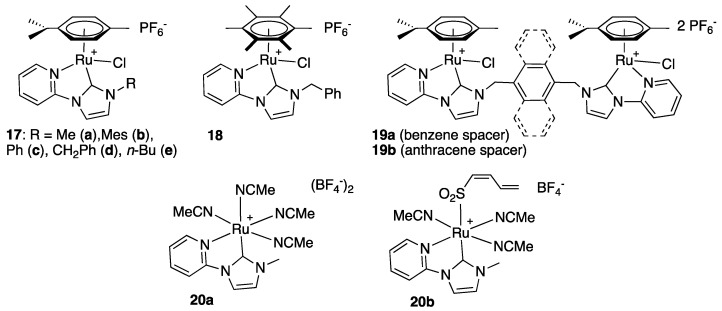
Pyridyl-functionalized imidazol-2-ylidene complexes **17**–**20**.

A number of η^6^-arene and octahedral ruthenium complexes bearing pyridyl-functionalized imidazole-2-ylidenes (Figure 3) was reported by several groups to catalyze the TH of acetophenone from isopropanol at reflux [28,29,30,31]. Relatively modest activities were observed in all cases as maximum TON ranged from 10 to 19 in the cases of **17a** and **20a**,**b** [28] and from 198 to 200 in the cases of **17d**, **18**, and **19a**,**b** [30], and as rather hard conditions were necessary to reach these values, i.e., *i*PrOH at 82 °C for 2 h with catalyst loadings as high as 5 mol% in the former case [28], and base (KOH or NaOH) loadings as high 1 equivalent in *i*PrOH at 80 °C for 2 h in the latter case [30]. Notably, complexes **17d**, **18**, and **19a**,**b** also reduced a couple of electron-deficient aromatic ketones with comparable efficiency. Electron-rich aromatic ketones required slightly longer reaction times (4–5 h instead of 1–2 h). The authors of this study proposed a classical monohydride mechanism that was checked by DFT calculation at the B3LYP level. Interestingly, all postulated intermediates could be optimized and were found to be energetically favorable [30]. Finally, it is worth mentioning that the activity of **17e** (1 mol%) in the presence of KOH (10 mol%) was compared to that of its Os, Cp*Rh, and Cp*Ir counterparts, and that it was found slightly more active than them [31].

**Scheme 12:**
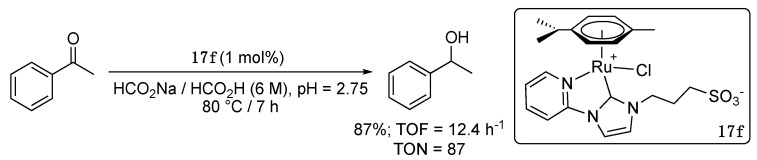
TH of acetophenone in aqueous solution catalyzed by the water soluble complex **17f**.

The water-soluble derivative **17f** of complex **17e**, bearing a propylsulfonate substituent instead of a *n*-butyl group, was prepared by transmetalation from the corresponding silver carbene complex and applied as catalyst for the TH of acetophenone in aqueous medium using HCO_2_Na/HCO_2_H as buffer (6 M, pH = 2.75) and hydrogen source [31]. A moderate yield of 87% was obtained after 7 h reaction (TOF = 12.4 h^−1^) at 80 °C with a catalytic charge of 1 mol% (Scheme 12). Notably, the Cp*Rh analogue proved much more active in this case, affording a 96% yield in only 10 min (TOF = 576 h^−1^) under these conditions.

**Scheme 13:**
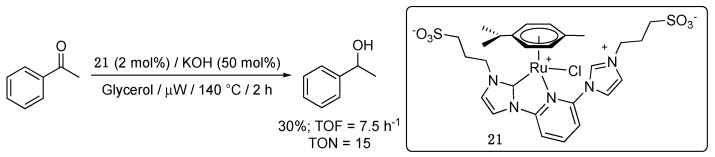
TH of acetophenone from glycerol catalyzed by the bis-sulfonated complex **21**.

In the same vein, the related pyridyl-functionalized imidazole-2-ylidene complex **21**, that bears two propylsulfonate groups, including one that is bound to a pendant imidazolium, was shown by Voutchkova-Kostal to be moderately active for the TH of acetophenone from glycerol, an inexpensive, renewable, non-toxic, and fully biodegradable solvent and hydrogen donor [32]. Thus, in glycerol at 140 °C under microwave heating, 30% yield to 1-phenylethanol was achieved after 2 h reaction with 2 mol% of **21** and 50 mol% KOH (Scheme 13).

**Figure 4:**
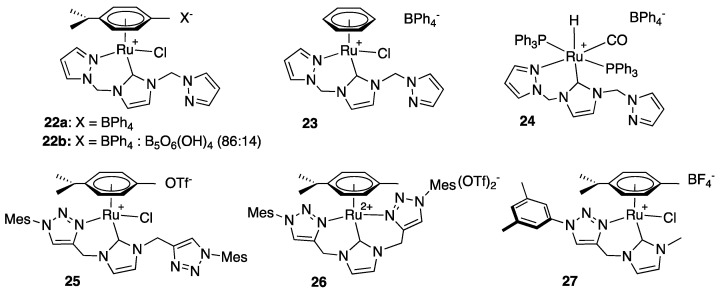
Triazole- and pyrazole-functionalized imidazole-2-ylidene complexes **22**–**27**.

Rare examples of pyrazole- and triazole-functionalized imidazole-2-ylidene-Ru(II) complexes were reported by the groups of Messerle [33], Kühn [34], and Rit [35]. Similarly to the pyridine-functionalized NHC complexes **17**–**20**, relatively modest activities were observed for the TH of acetophenone with the pyrazole derivatives **22a**, **23**, and **24** that bear both a coordinated and an uncoordinated pyrazole moiety (Figure 4). They indeed required 4 h reaction at reflux with catalyst loadings of 1.5 mol% and base loadings of 18 mol% (KOH) to achieve conversions of 80% (**22a**: TOF = 13.3 h^−1^; TON = 53), >99% (**23**: TOF = 16.6 h^−1^; TON = 66.7) and 24% (**24**: TOF = 4 h^−1^; TON = 16) [33]. The very modest activity observed with the hydride complex **24**, in particular, was surprising, as the pre-existing hydride ligand was expected to enable a more efficient TH reaction. The triazole derivatives **25**, **26**, and **27** proved slightly more active with maximum TON of 170, 168, and 500, respectively, under milder conditions, i.e., 0.1 (**27**) to 0.5 (**25**, **26**) mol% catalyst and only 5 (**25**, **26**) to 10 (**27**) mol% NaO*i*Pr or KO*t*Bu as base in isopropanol at reflux for 2–3 h [34,35]. It is noteworthy, however, that full conversion could not be attained in the cases of **25** and **26** under these conditions. Thus, although the tridentate complex **26**, with both triazole moieties coordinated to the ruthenium center, exhibits the highest TOF of these two triazole derivatives, with a TOF as high as 1100 h^−1^ at t = 2 min (19% conversion), it slows down significantly after 60% conversion to reach a maximum conversion of 84% after 2 h reaction (TOF = 84 h^−1^), suggesting an instability of the active species. Unfortunately, no mechanism insight was provided.

**Scheme 14:**
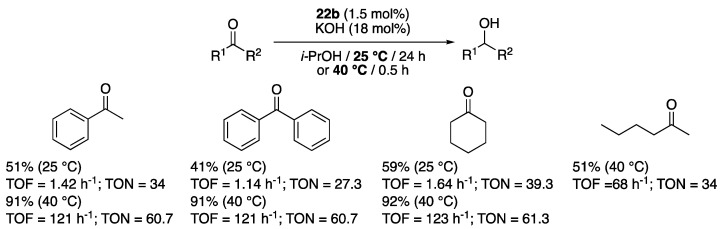
Low-temperature TH of ketones catalyzed by the anion-mixed pyrazole-imidazol-2-ylidene complex **22b**.

The mixed anion complex **22b**, that contains an unusual 86:14 mixture of BPh_4_^−^ and [B_5_O_6_(OH)_4_]^−^ as a counter-anion deserves a particular attention. It indeed proved much more active than **22a** containing pure BPh_4_^−^, and was demonstrated to be a very rare example of Ru(II) complex able to catalyze the TH of various ketones at low temperatures (25 to 40 °C, Scheme 14) [33] (see the works of Bera [36], Sakaguchi [37], and Pfeffer and de Vries [38,39] for other rare known examples of low-temperature efficient Ru(II)-based TH catalysts). The difference of activity between **22a** and **22b**, suggests a non-innocent role of the pentaborate anion, which most certainly acts as a catalyst in its own right, as has been shown in the literature for various polyborate anions [40,41].

**Scheme 15:**
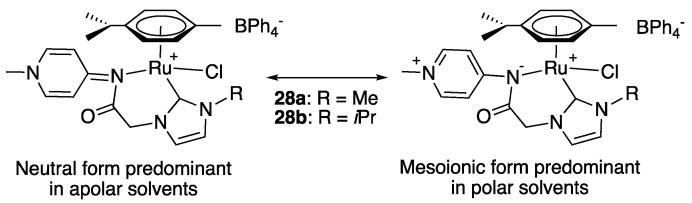
Solvent-dependent resonance forms of the pyridylideneamide-imidazol-2-ylidene-Ru(II) complexes **28a**,**b**.

In 2014, Albrecht and Wright introduced an interesting class of hybrid pyridylideneamide-imidazol-2-ylidene ligands that has two dissimilar strong σ donors, comprised of a relatively soft C donor with significant covalent bonding preference and of a harder N donor that favors ionic interactions [42]. Interestingly, the resulting half-sandwich ruthenium (II) complexes **28a**,**b**, which were prepared by transmetalation of the adequate hybrid ligand from silver to [Ru(η^6^-*p*-cymene)Cl_2_]_2_, displayed two solvent-dependent limiting resonance structures. While the neutral pyridylidene imine form was shown by cyclic voltammetry, NMR and UV-visible spectroscopy to be predominant in apolar solvents such as dichloromethane, the mesoionic pyridinium amidate form was found predominant in polar solvents such as methanol and DMSO (Scheme 15). This property was demonstrated to have a direct influence on the catalytic activity of **28a** for the dehydrogenation of benzyl alcohol to benzaldehyde in various solvents, the best results being observed in the very polar DMSO. Nevertheless, both **28a** and **28b** (5 mol%, 10 mol% KOH) behaved as very poor catalysts for the TH of benzophenone to diphenylethanol in isopropanol at reflux, with maximum conversions of 20% (TON = 4) after 6 h (TOF = 0.67 h^−1^), presumably because of a massive decomposition of the complexes under these conditions, as shown by the presence of black particles in the medium at the end of the reactions [42].

**Scheme 16:**
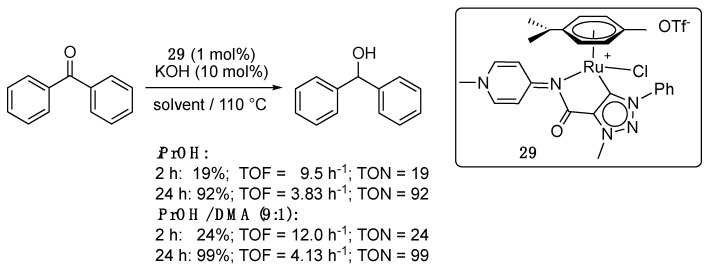
Solvent dependence of the TH of benzophenone catalyzed by the adaptive pyridylideneamide-triazolylidene complex **29**.

More recently, Albrecht completed his study with the synthesis, via a similar transmetalation route, and catalytic investigation in TH of the complex **29** bearing a hybrid pyridylideneamide-triazolylidene ligand [43,44]. The latter achieved 92% reduction in benzophenone in 24 h with 1 mol% loading (TOF = 3.83 h^−1^) in the presence of 10 mol% KOH in refluxing isopropanol (Scheme 16). Interestingly, increasing the polarity of the reaction medium by the addition of 10% dimethylacetamide (DMA) as a co-solvent to isopropanol had a notable impact on the catalytic activity of **29** [43]. Under these conditions, it achieved essentially full conversion after 24 h, and 24% conversion after 2 h (vs. 19% in pure isopropanol, Scheme 16). This increased activity in a more polar medium was attributed to an increased relevance of the mesoinonic resonance form. Furthermore, no decomposition was observed with **29** under the basic conditions of the TH reaction. This enhanced stability of the triazolylidene derivative **29** under basic conditions when compared to the imidazol-2-ylidene derivatives **28a**,**b** was attributed to the absence of an acidic CH_2_ group between the pyridylideneamide and the triazolylidene [43].

**Scheme 17:**
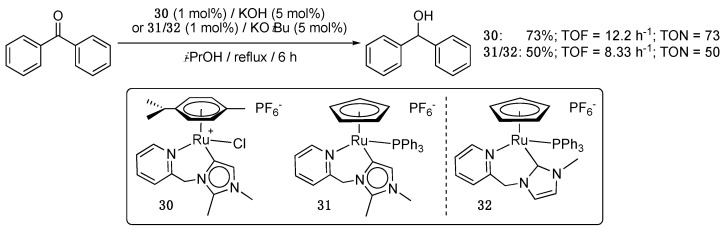
TH of benzophenone catalyzed by the pycolyl-functionalized imidazol-4-ylidene complexes **30** and **31**/**32**.

Albrecht and Landman recently reported the synthesis and catalytic activity in ketone TH of rare examples of picolyl-functionalized imidazol-4-ylidene ruthenium(II) complexes [45]. C(4)/C(5)-bound imidazolylidenes are known to be significantly stronger donors compared to their C(2)-bound counterparts [46]. Their synthetic accessibility is however limited by the low acidity of the imidazolium C(4)/C(5) protons, which requires specific protocols to achieve metalation at this position, such as the protection of the C(2)-position with alkyl or aryl groups [47] or the chelation assistance strategy [48]. The authors used a combination of these two strategies to prepare η^6^-*p*-cymene and η^5^-cyclopentadienyl (Cp) derivatives bearing a 1,2-dimethyl-3-pycolyl-imidazol-4-ylidene ligand. While the *p*-cymene complex **30** was isolated in a pure form, the Cp derivative **31** was isolated as a 1:1 mixture with the 1-methyl-3-pycolyl-imidazol-2-ylidene complex **32** resulting from C(2)–CH_3_ bond cleavage (Scheme 17). Both complexes (1 mol%) showed a relatively moderate activity with initial TOF values measured after 15 min of reaction of 34 h^−1^ (**30**) and 22 h^−1^ (**31**/**32**), and TON of 73 (**30**) and 50 (**31**/**32**) after 6 h reaction in isopropanol at reflux with a base loading of 5 mol%. Interestingly, closely related derivatives of **30** and **31** bearing a *N*-2-methylprop-2-ene tether instead of a *N*-picolyl group displayed slightly higher activity, notably in the case of the *p*-cymene derivative, which showed an initial TOF of 60 h^−1^ and a TON of 93 after 6 h. The higher activity observed with the *p*-cymene complexes was attributed to an easier substitution by the base of the chloride ligand in the *p*-cymene complexes than of any of the ligands in the Cp complexes.

Interestingly, complexes **30** and **31**/**32** proved to be also moderately active in the anaerobic oxidation of 1-phenylethanol to acetophenone in the presence of KO*t*Bu (5 mol%) in *o*-dichlorobenzene at 150 °C, with conversions of 73 and 56% after 24 h reaction, respectively [45]. As no hydride signals of either the *p*-cymene or Cp catalysts could be observed by ^1^H NMR spectroscopy in both the TH and alcohol oxidation reactions, the authors proposed a monohydride mechanism where the monohydride intermediate catalytic species would not be the resting-state intermediate species.

**Figure 5:**
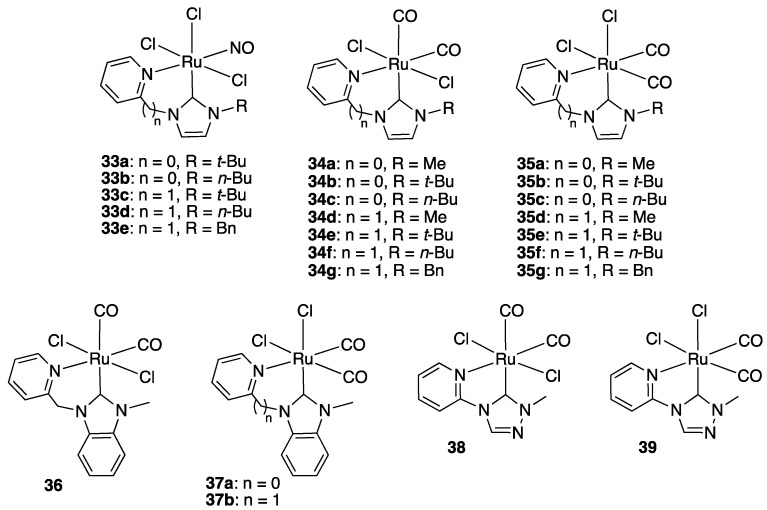
Pyridyl- or picolyl-functionalized imidazol-, benzimidazol- or triazol-2-ylidene octahedral Ru(II) complexes **33**–**39**.

Aside from all these half-sandwich complexes, a number of octahedral complexes were also reported as ketone TH catalysts, notably a series of ruthenium(II) nitrosyl trichloride (**33a**–**e**) and dicarbonyl dichloride (**34**–**39**) complexes bearing pyridyl- or picolyl-functionalized imidazol-, benzimidazol-, or triazol-2-ylidenes (Figure 5) [49,50,51]. With the exception of the picolyl-benzimidazol-2-ylidene complex **37a**, which was synthesized by direct reaction of the corresponding benzimidazolium hexafluorophosphate with [Ru(CO)_2_Cl_2_] in the presence of NEt_3_ [51], they were all prepared by transmetalation from the adequate in situ prepared silver carbene complex with either [Ru(NO)Cl_2_] or [Ru(CO)_2_Cl]_n_. The observed activity in TH decreased in the order of the σ-donor ability of the NHC ligand: imidazolylidene (**33a**–**35g**) > benzimidazolylidene (**36**–**37b**) > triazolylidene (**38**, **39**). Among the imidazol-2-ylidene derivatives, the methyl- and *t*-butyl-substituted complexes **33a**, **33c**, **34a**, **35a**, and **35b** proved to be the most active. With the exception of the nitrosyl species **33c**, all these were comprised of a five-membered ring. In particular, the 1-methyl-3-pyridyl-imidazol-2-ylidene complex **34a** showed the highest TOFs (from 87.5 to 248 h^−1^ with a small range of 6 aromatic and aliphatic ketones), and the 1-*t*-butyl-3-pyridyl-imidazol-2-ylidene derivative **35b**, which could work with a loading as low as 0.2 mol% in the presence of 5 mol% KOH in isopropanol at 82 °C, the highest TONs (from 50 to 455).

**Scheme 18:**
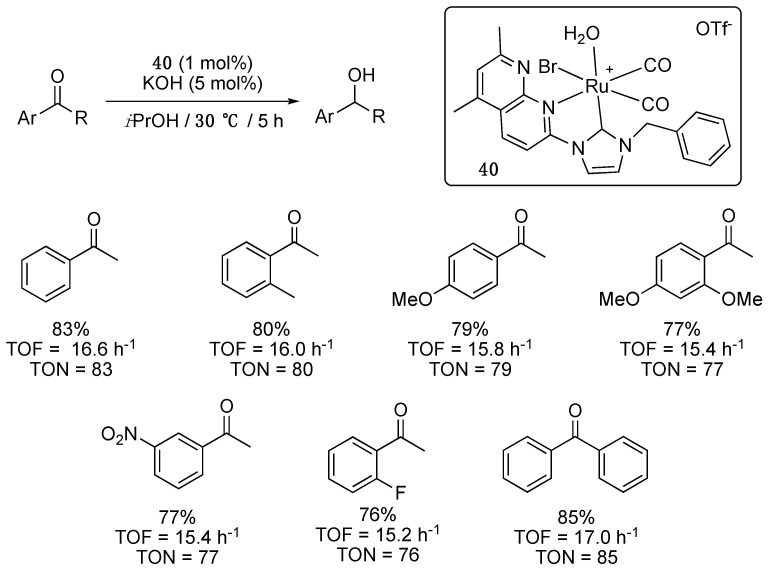
Low-temperature TH of aromatic ketones catalyzed by the aqua-Ru(II) complex **40** bearing a 1,8-naphtyrid-2-yl-imidazol-2-ylidene chelate.

Room temperature treatment of [Ru_2_(CO)_4_(CH_3_CN)_6_](OTf)_2_ with two equivalents of 1-benzyl-3-(5,7-dimethyl-1,8-naphtyrid-2-yl)imidazolium bromide provided the related octahedral aqua-bromo-dicarbonyl-ruthenium(II) complex **40** which was reported by Bera et al. to act as a rare example of low-temperature-efficient catalyst for the TH of a short series of aromatic ketones in the presence of 5 mol% KOH [36]. With a catalyst loading of 1 mol%, TON ranging from 76 to 85 were achieved in 5 h (TOF = 15.2–17 h^−1^) at 30 °C with electron-neutral, as well as electron-poor and electron-rich aromatic ketones (Scheme 18). Heteroaromatic ketones such as acetylthiazole and acetylpyridine, however, required 12 h at 80 °C to reach moderate yields: 65% (TOF = 5.41 h^−1^) and 59% (4.92 h^−1^), respectively. No conversion was observed for acetylpyrrole even after prolonged heating.

**Scheme 19:**
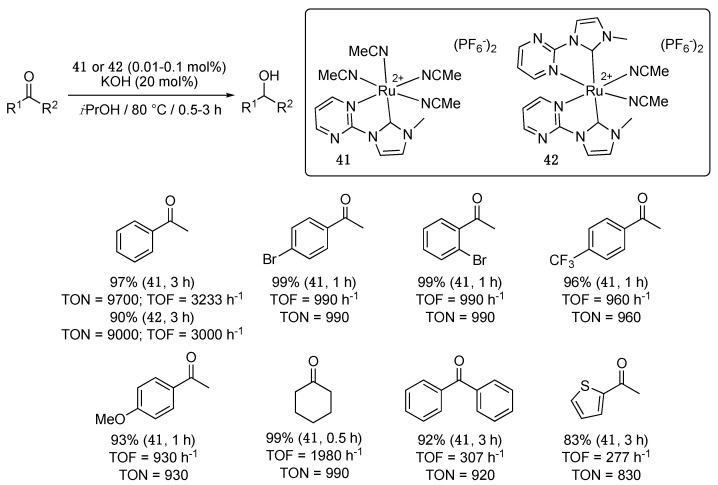
TH of ketones catalyzed by the pyrimidin-2-yl-functionalized imidazol-2-ylidene complexes **41** and **42**.

Finally, a pyrimidine-functionalized imidazol-2-ylidene-Ru(II) motif similar to that found in the *p*-cymene complex **2** was used in the octahedral dicationic complexes **41** and **42** reported by W. Chen et al. in 2015 [52]. The complexes bearing either one or two chelating units and, respectively, four or two acetonitrile ligands were obtained by transmetalation from the corresponding nickel-NHC complexes with 0.5 or 0.25 equiv. of [Ru(η^6^-*p*-cymene)Cl_2_]_2_ in acetonitrile at reflux. Both complexes showed greatly improved catalytic performances compared to the half-sandwich complex **2**, and to the related octahedral complexes **9b** and **13b**, bearing a pyridyl- or pycolyl-functionalized triazolylidene and four acetonitrile ligands, with TON and TOF values for the TH of acetophenone as high as 9700 and 3233 h^−1^ (**41**) and 9000 and 3000 h^−1^ (**42**) (Scheme 19). Much more basic conditions than with **2**, **9b**, and **13b** were however required; 20 mol% KOH vs. 10 mol% with **2**, **9b**, and **13b**.

A brief study of the reaction scope, carried out with 0.1 mol% **41**, showed that it was very active in the TH of cyclohexanone and aromatic ketones bearing either electron-withdrawing or electron-donating substituents. Longer reaction times were however required with benzophenone and 2-acetylthiophene to observe high reduction yields (Scheme 19).

### 2.2. Ketones’ Transfer Hydrogenations with (κ^2^-C_NHC_,P)-Complexes

Examples of (*κ*^2^-*C_NHC_*,*P*)-complexes as ketone TH catalysts are surprisingly rare and involve mainly abnormal NHC-phosphine complexes, described by the group of Baratta. The first examples, however, arose from the groups of Grotjahn and Domsky in 2011 and 2013, and involved protic and classic NHCs, respectively.

**Scheme 20:**
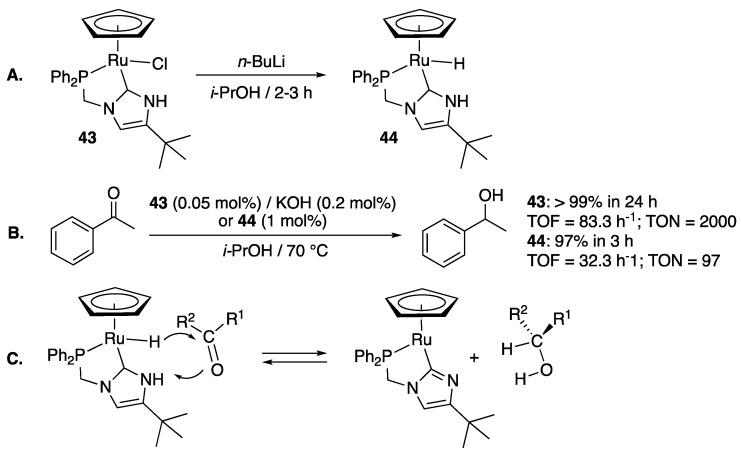
Synthesis of the hydride complex **44** from the protic NHC complex **43** (**A**); TH of acetophenone catalyzed by **43** and **44** (**B**); Proposed TH mechanism (**C**).

In the first example, Grotjahn et al. thus described the synthesis—via direct reaction of the corresponding imidazole with [RuCpCl(cod)] (cod = 1,5-cyclooctadiene) in THF at 100 °C—and reactivity of the CpRu complex **43** bearing a diphenylphosphino-functionalized protic imidazol-2-ylidene ligand [53]. Its reaction with *n*-BuLi in isopropanol provided the corresponding hydride complex **44** (Scheme 20A). Both complexes proved active for the TH of acetophenone (2 M) to 1-phenylethanol in isopropanol at 70 °C. Significantly, **44** (1 mol%) was efficient in the absence of a base and achieved 97% yield in 3 h, whereas **43** (0.050 mol%) required the presence of KOH (0.2 mol%) to produce **44** in situ and achieve full reduction in 24 h (Scheme 20B). The catalytic process is believed to involve the N-H function of the protic NHC as shown in Scheme 20C, and thus constitute a rare example of a cooperative hydrogenation–dehydrogenation process involving both a metal center and one ring nitrogen atom of a NHC ligand [54].

**Scheme 21:**
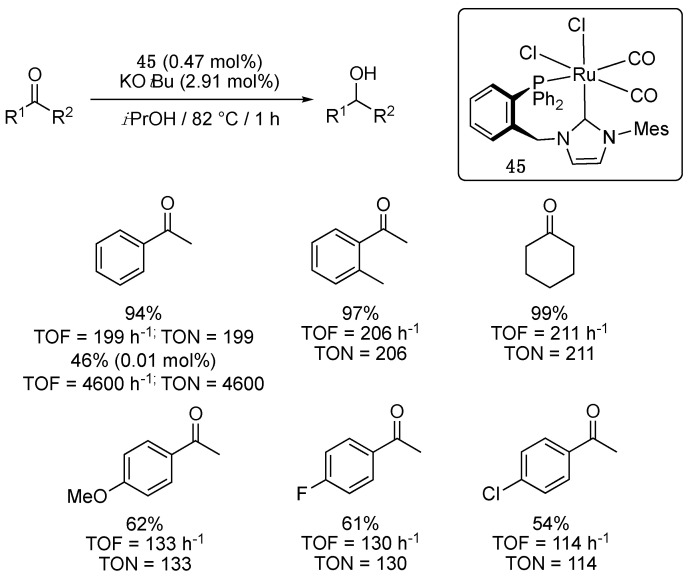
TH of ketones catalyzed by the *ortho*-(diphenylphosphino)benzyl-functionalized imidazol-2-ylidene complex **45**.

Two years later, Domski et al. reported the synthesis via a classical transmetalation procedure from silver of an octahedral ruthenium dicarbonyl dichloride complex **45**, bearing an *ortho*-(diphenylphosphino)benzyl-functionalized imidazol-2-ylidene ligand, that proved catalytically effective for the TH of a small number of ketones in refluxing isopropanol [55]. With a loading of 0.47 mol% in the presence of KO*t*Bu (2.91 mol%) as base, acetophenone, 2-methylacetophenone, and cyclohexanone were all almost fully reduced in 1 h, giving TOF ranging from 199 to 211 h^−1^ (Scheme 21). A TOF of 4600 h^−1^ could even be reached for acetophenone when a catalytic loading of 0.01 mol% was used under otherwise similar conditions. Substitution at the *para*-position, however, led to lower yields with both electron-donating and electron-withdrawing substituents.

**Scheme 22:**
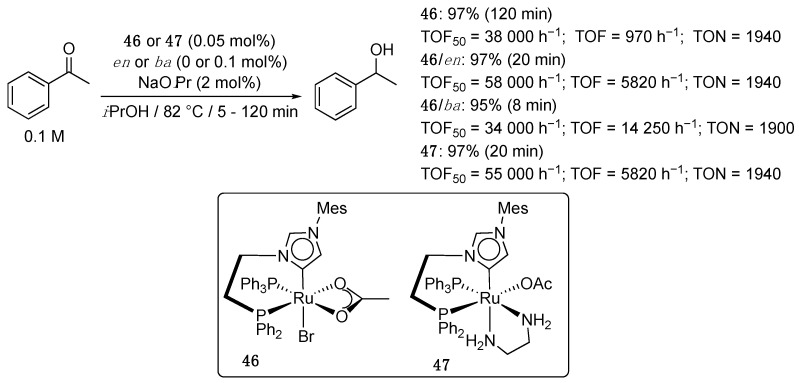
TH of acetophenone catalyzed by the (diphenylphosphino)ethylene-functionalized imidazol-4-ylidene complexes **46** and **47**.

At about the same period, Baratta et al. reported the first of a series of four articles on ruthenium(II) complexes bearing a bifunctional phosphine-imidazol-4-ylidene ligand [56]. In this initial account, the [RuBr(κ^2^-OAc)(PPh_3_)(Ph_2_P-(CH_2_)_2_-aNHC)] complex **46** was prepared by treatment of [Ru(OAc)_2_(PPh_3_)_2_] with 1.2 equivalents of the corresponding phosphine-imidazolium bromide in the presence of sodium acetate in THF under reflux. Complex **46** (0.05 mol%) showed high initial activity for the TH of acetophenone (0.1 M) to 1-phenylethanol in refluxing 2-propanol with a TOF_50_ as high as 38,000 h^−1^, but deactivated quickly to reach full conversion after 2 h and a final TOF of only 970 (Scheme 22), which was still about five times higher than the TOF observed with complex **45**. The addition of ethylenediamine (*en*) or benzylamine (*ba*) (0.1 mol%) as nitrogen ligands containing a N–H function [57] gave more active and productive catalysts. Thus, in the presence of these ligands, acetophenone was reduced quantitatively in 20 or 8 min, respectively, leading to TOF of 5820 and 14 250 h^−1^. Interestingly, the isolated complex, [Ru(κ^1^-OAc)(PPh_3_){Ph_2_P-(CH_2_)_2_-aNHC}{H_2_N-(CH_2_)_2_-NH_2_}] (**47**), showed the same activity as the in situ generated system, **46**/*en*, and underwent fast proton exchange of the four NH and the carbene NCHN protons in basic D_2_O, unveiling a possible active role in bifunctional catalysis [57]. The study was completed by a brief overview of the reaction scope that revealed that hex-5-en-2-one, 2-methylcyclohexanone and benzophenone could all be fully and selectively reduced to the corresponding alcohols in 2 to 60 min in the presence of **46**/*en* under similar reaction conditions.

**Scheme 23:**
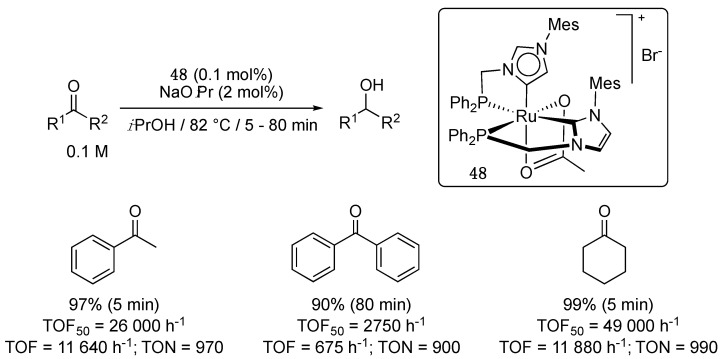
TH of ketones catalyzed by the (diphenylphosphino)methylene-functionalized imidazol-2-ylidene and imidazol-4-ylidene complex **48**.

In 2015, the same group described a rare example of ruthenium *bis*-carbene complex **48** bearing both a (diphenylphosphino)methylene-functionalized imidazol-2-ylidene and imidazol-4-ylidene ligand, that may form on account of steric factors involving the bulky mesityl substituents carried by the *N*-heterocyclic carbenes [58]. It could be prepared either in one or two steps by the reaction of [Ru(OAc)_2_(PPh_3_)_2_] with 1 + 1 or directly 2 equivalents of the corresponding phosphine–imidazolium bromide in the presence of sodium acetate in THF at reflux. Catalytic studies revealed high activities for the TH of a couple of ketones with TOF_50_ and final TOF values of up to 49,000 h^−1^ and 11 880 h^−1^, respectively (Scheme 23) [58,59]. These values make of **48** a TH catalyst of comparable activity to **46**/*en* [56], although it does not comprise an N–H function, which often entails high activity through bifunctional catalysis [57].

Derivatization of complexes **46** and **48** by coordination of Ag or of an IrCl(η^4^-cyclooctadiene) fragment to the abnormal C2 carbon generated bimetallic TH catalysts that displayed much lower activity than **46** and **48** [59], indirectly suggesting that the NCHN proton of the imidazol-4-ylidene ligand could play an active role in the TH catalysis, in agreement with the fast proton exchange observed with **47** in basic D_2_O (*vide supra*) [56].

**Scheme 24:**
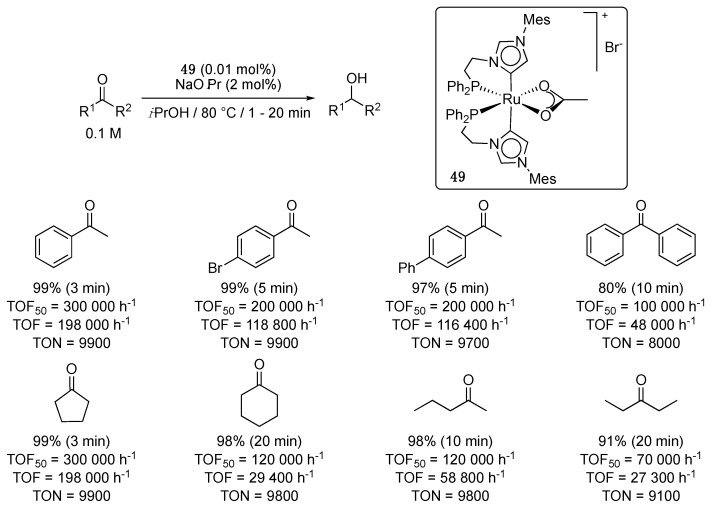
TH of ketones catalyzed by the (diphenylphosphino)methylene-functionalized bis-imidazol-4-ylidene complex **49**.

More recently, Baratta et al. disclosed the synthesis and characterization of the first ruthenium complex bearing two abnormal NHC ligands, **49** [60]. This (diphenylphosphino)ethylene-functionalized bis-imidazol-4-ylidene species, which was synthesized in 85% yield by reaction of complex **46** with two equivalents of 1-(diphenylphosphino)ethylene-3-mesitylene-imidazol-2-ylidene in the presence of sodium acetate as base in THF at 60 °C, displayed outstanding catalytic activity in both ketone TH and the reverse Oppenauer-type alcohol oxidation. At 80 °C in isopropanol, in the presence of NaO*i*Pr (2 mol%), acetophenone was quantitatively reduced to 1-phenylethanol within 3 min using a catalytic loading of 0.01 mol%, giving TOF_50_ and TOF at full conversion of 300,000 and 198,000 h^−1^, respectively (Scheme 24). Even more impressively, at reflux, the reduction occurred in only 1 min, affording a TOF_50_ of 600,000 h^−1^, which is among the highest TOFs reported in TH [61], and makes of **49** the most active Ru-carbene TH catalyst to date [56,59,62,63,64]. 4-Bromo-acetophenone, 4-acetylbiphenyl, cyclopentanone, cyclohexanone and 2-pentanone were all fully reduced within 3 to 20 min with the same catalytic loading, giving TOF at full conversion ranging from 29 400 h^−1^ and 198,000 h^−1^ (Scheme 24). Benzophenone was converted to benzhydrol in 80% in 10 min (TOF = 48,000 h^−1^, TON = 8000) and 3-pentanone to 3-pentanol in 91% in 20 min (TOF = 27 300 h^−1^, TON = 9100).

**Scheme 25:**
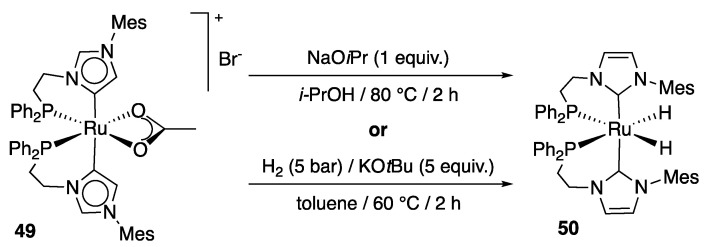
Formation of the dihydride bis-imidazol-2-ylidene complex **50** from **49**.

Interestingly, VT NMR experiments revealed that a rapid H/D exchange occurred at the NCHN proton positions of the two imidazole-4-ylidene ligands when NaO*i*Pr (1 equiv.) was added to **49** in 2-propanol-*d*_8_ at RT, similarly to what had been observed with complex **46** in basic D_2_O (*vide supra*) [56]. In addition, the reaction of **49** with NaO*i*Pr (10 equiv.) in 2-propanol at 80 °C for 2 h led to the formation of the dihydride complex **50** bearing two (diphenylphosphino)methylene-functionalized imidazol-2-ylidenes (Scheme 25). The latter can also be obtained by reaction of **49** with H*_2_* (5 bar) in the presence of KO*t*Bu in toluene at 60 °C. Complex **50** proved, however, to be inactive in catalytic TH. The abnormal carbene coordination found in **49** is therefore considered by the authors to be crucial to observe high catalytic performances, on account of the smaller steric hindrance of the mesityl groups in this species, and possibly via deprotonation at the C2 carbene carbons [60]. Accordingly, the formation of **50** is regarded as a catalyst deactivation pathway.

### 2.3. Ketones’ Transfer Hydrogenations with (κ^2^-C_NHC_,O)-Complexes

Although the first report appeared a decade ago [65], examples of (*κ*^2^-*C_NHC_*,*O*)-complexes as ketone TH catalysts remain very rare and, with one exception, always comprise (benz)imidazol-2-ylidene or 1,2,3-triazolylidene ligands functionalized with a carboxylate side-arm.

**Scheme 26:**
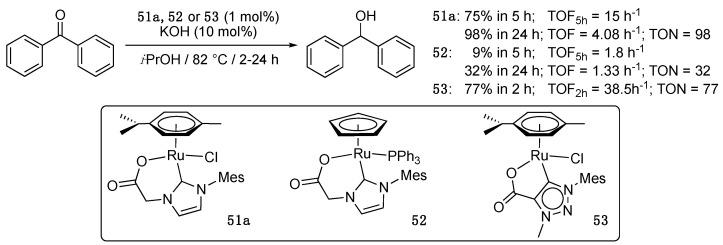
TH of benzophenone catalyzed by the carboxylate-functionalized imidazol-2-ylidene and 1,2,3-triazolylidene complexes **51a**, **52** and **53**.

Thus, the first examples of this short series were reported in 2011 by Albrecht et al. with the description of the catalytic activity in the TH of benzophenone of two carboxylate-functionalized imidazole-2-ylidene complexes, **51a** and **52**, that bore the same functionalized carbene but differed by the nature of the ruthenium scaffold, “(η^6^-*p*-cymene)RuCl” vs. “CpRu(PPh_3_)”, and were prepared in a one-pot procedure via transmetalation from the corresponding silver carbene complex to [RuCl_2_(η^6^-*p*-cymene)]_2_ or [RuCpCl(PPh_3_)_2_], respectively [65,66]. Whereas the (η^6^-*p*-cymene)RuCl derivative **51a** achieved full conversion in 24 h in refluxing 2-propanol with a loading of 1 mol% and 10 mol% of KOH as activator, the CpRu(PPh_3_) derivative **52** displayed very poor activity, reaching only 32% conversion after 24 h under the same conditions (Scheme 26). These two initial half-sandwich *C*,*O*-catalysts were joined a couple of years later by a novel (η^6^-*p*-cymene)RuCl derivative **53** bearing a mesoionic carbene C-functionalized with a carboxylate group [23]. The latter proved to be more than twice as reactive as **51a**, achieving 77% conversion in 2 h under the same conditions, while **51a** necessitated 5 h to reach a similar conversion (Scheme 26).

**Figure 6:**
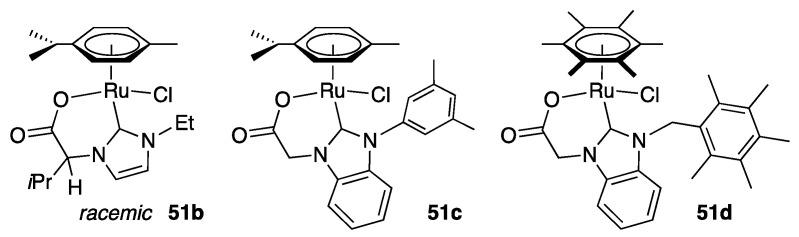
Carboxylate-functionalized NHC-Ru(II) complexes **51b**–**d**.

At the same period, Papish [67], Tennyson [68], and Altin and coworkers [69] described the syntheses, via similar means, and catalytic activities of complexes **51b**, **51c**, and **51d**, respectively (Figure 6), which closely resemble **51a**. Noteworthy, although **51b** was prepared from the corresponding enantiopure proligand (in the form an ester), it was obtained as a racemic mixture. The activity of all three complexes were evaluated for the TH of acetophenone from isopropanol at reflux. Complex **51d** proved to be most active of the three, as it achieved 95% conversion in 2 h with a catalyst loading of 0.4 mol% (and 10 mol% KO*t*Bu), giving a TON of 238 and a TOF of 119 h^−1^ [69]. In contrast, complexes **51b** (0.5 mol% with 12.5 mol% KOH) and **51c** (1 mol% with 4.95 mol% KO*t*Bu) only achieved initial TOF_1h_ of 44 and 31 h^−1^, and TON of 190 and 96 after 6 and 24 h reaction, respectively [67,68]. However, **51b** showed some inconsistency with anomalous conversions as high as 88% after 1 h, which would correspond to an initial TOF_1h_ of 176 h^−1^ [67]. Papish et al. suggested that this could be due to decomposition to a highly active heterogeneous catalyst or to a reaction pathway that would be highly sensitive to trace contaminants, such as a radical mechanism, but no mechanistic investigations were carried out to sustain these assumptions.

**Scheme 27:**
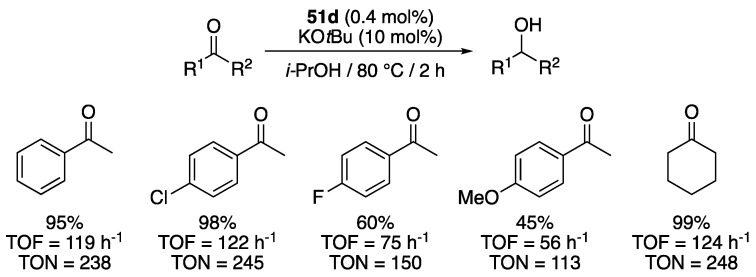
TH of ketones catalyzed by the carboxylate-functionalized benzimidazolylidene complex **51d**.

Altin et al. briefly examined the scope of substrates that could undergo TH by the carboxylate-functionalized benzimidazolylidene complex **51d** under the established conditions (0.4 mol% **51d**, 10 mol% KO*t*Bu, *i*PrOH at 80 °C) [69]. Similarly to acetophenone, *p*-chloroacetophenone and cyclohexanone, were converted to the corresponding alcohols in nearly quantitative yield in 2 h (Scheme 27). *p*-Fluoroacetophenone and *p*-methoxyacetophenone gave lower TOF, but still achieved TON of over 110 in 2 h.

**Scheme 28:**
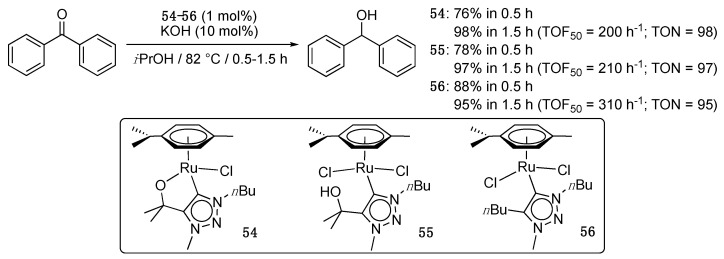
TH of benzophenone catalyzed by the hydroxy-functionalized 1,2,3-triazolylidene complexes **54** and **55** and the 1,4-dibutyl-triazolylidene complex **56**.

A last example deals with hydroxy-functionalized 1,2,3-triazolyildene complexes, in which the pendant hydroxyl group can be either chelating (**54**) or not (**55**) [70]. The monodentate and bidentate bonding modes are interconvertible by the addition of a chloride scavenger such as AgBF_4_ or NH_4_BF_4_, or conversely brine, which demonstrates the hemilabile character of the hydroxyl group. Both complexes (1 mol%) reached 76–78% conversion in 0.5 h and full conversion in 1.5 h for the TH of benzophenone in the presence of KOH (10 mol%) in isopropanol at reflux (Scheme 28). The activity of the analogous complex **56** bearing a 1,4-dibutyl-1,2,3-triazolylidene was also evaluated for comparison. It reached the 78% conversion in 15 min and 88% conversion in 0.5 h. The higher activity of the latter (TOF_50_ = 310 h^−1^ vs. 200 and 210 h^−1^ for **54** and **55**, respectively) clearly shows the absence of beneficial role of the hydroxy group, whether it is chelating or not. As stated by the authors, these data militate in favor of an inner sphere monohydride mechanism [4], in which the intermediate ruthenium-hydride species is formed via β-H-elimination from the previous ruthenium-isopropoxide species without any help of the hydroxy-functionality, in contrast to what was initially hoped.

## 3. Asymmetric Transfer Hydrogenation (ATH) of Ketones

To the best of our knowledge, donor-functionalized NHC-ruthenium complexes have, surprisingly, almost never been used in ATH of ketones. We are indeed aware of only two reports of chiral catalysts, including one that gave almost no enantiomeric excess (*ee*).

### 3.1. Ketones’ Asymmetric Transfer Hydrogenation with (κ^2^-C_NHC_,N)-Complexes

**Scheme 29:**
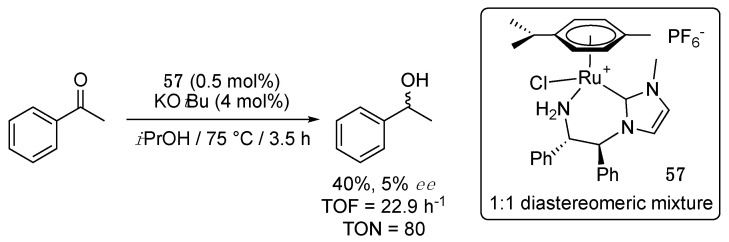
ATH attempt of acetophenone with the (*S*,*S*)-1,2-diphenylethylamine-functionalized imidazol-2-ylidene complex **57**.

In 2016, Morris et al. disclosed an asymmetric version of their cationic amino-functionalized imidazol-2-ylidene complex **4a** [14]. The chiral complex **57**, bearing a (*S*,*S*)-1,2-diphenylethylamine-functionalized imidazol-2-ylidene, was obtained as a 1:1 diastereomeric mixture with opposite chirality at the ruthenium center by transmetalation from silver [71]. In isopropanol at 75 °C, **57** (0.5 mol%), along with 4 mol% KO*t*Bu, afforded 40% conversion of acetophenone to 1-phenylethanol after 3.5 h of reaction (TOF = 22.9 h^−1^, TON = 80), with an enantioselectivity of only 5% (Scheme 29). The poor activity compared to **4a** (TOF = 216 h^−1^, TON = 1080 in 5 h) was attributed to the low solubility of **57** in isopropanol, and the low enantioselectivity to its 1:1 diastereomeric composition or, tentatively, to an inner-sphere mechanism involving the decoordination of the amine side arm, as was proposed for **4a** [14]. Of note, **57** (0.5 mol% with 4 mol% KO*t*Bu) also showed moderate direct hydrogenation activity under 25 bar H_2_ at 50 °C in THF (TOF = 200 h^−1^, TON = 200), but no enantioselectivity at all in this case.

### 3.2. Ketones’ Asymmetric Transfer Hydrogenation with (κ^2^-C_NHC_,O)-Complexes

**Scheme 30:**
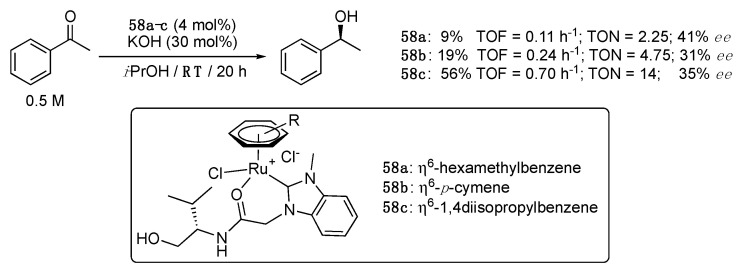
ATH of acetophenone with the hydroxy-amide-functionalized benzimidazol-2-ylidene (η^6^-arene)Ru(II) complexes **58a**–**c**.

**Scheme 31:**
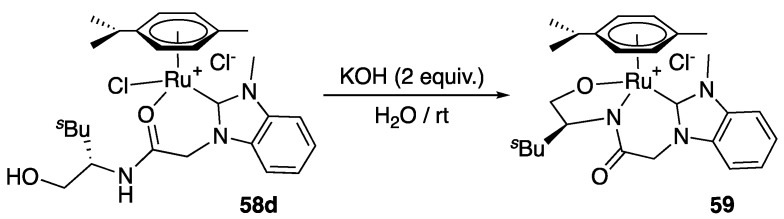
Synthesis of the catalytically inactive dianionic alkoxy-amidate-NHC-Ru complex **59**.

In 2013, Sakaguchi et al. prepared via transmetalation from silver to the corresponding [RuCl_2_(η^6^-arene)]_2_ precursor a series of benzimidazol-2-ylidene (η^6^-arene)Ru(II) complexes **58a**–**c** bearing a hydroxy-amide chelating side arm, and applied it to the ATH of acetophenone [37]. All complexes acted as precatalysts at room temperature with low to moderate activity and relatively low enantioselectivity (Scheme 30). Interestingly, the nature of η^6^-arene ligand was shown to significantly alter the activity and selectivity of these complexes, with the η^6^-hexamethylbenzene derivative **58a** giving the highest enantioselectivity (41% *ee*) and the η^6–^1,4-(diisopropyl)benzene complex **58c** the highest activity (TOF = 0.70 h^−1^ after 20 h vs. 0.11 and 0.24 h^−1^ for **58a** and **58b**). Furthermore, the hydroxy functional group on the NHC side arm was found to be of critical importance, as its replacement by a methoxy group resulted in no activity. However, use of the dianionic alkoxy-amidate-NHC-Ru complex **59**, obtained by treating the complex **58d** with KOH at room temperature (Scheme 31), resulted in no activity in acetophenone TH, showing the probable importance of having a possible vacant site to which the reacting ketone can coordinate.

## 4. Transfer Hydrogenation of Aldehydes

The TH of aldehydes is rarely studied, and the catalytic efficiency is generally found to be low with limited substrate scope along with the formation of aldol condensation or carboxylic acid byproducts [5]. We are, thus, aware of only a small number of (*κ*^2^-*C_NHC_*,*N*)-complexes as precatalysts for this transformation.

Aldehydes TH with (*κ*^2^-*C_NHC_*,*N*)-Complexes

**Scheme 32:**
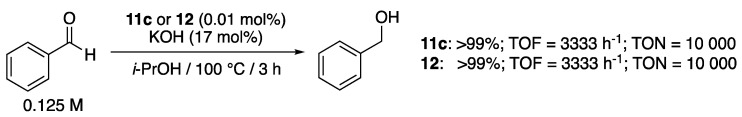
TH of benzaldehyde catalyzed by the pyridine- or pyrimidine-functionalized triazolylidene complexes **11c** and **12**.

The pyridine- or pyrimidine-functionalized triazolylidene complexes **11c** and **12** (0.01 mol%), described by Sarkar et al. [24,25], showed exceptional activities for the TH of benzaldehyde to benzylic alcohol in isopropanol at reflux in the presence of KOH (17 mol%), both achieving TOF of 3333 h^−1^ and TON of 10,000 (Scheme 32). Remarkably, no aldol-type condensation product was observed. This is in sharp contrast to what was observed by Albrecht et al. with the related pyridyl-functionalized triazolylidene complex **9a**, that mainly differs from **11c** and **12** by the bonding of the pyridyl unit to the triazole carbon instead of the triazole nitrogen, and with which double aldol condensation products were formed predominantly during the attempted TH of aldehydes such as benzaldehyde, *p*-chlorobenzaldehyde and thiophenyl aldehyde under similar conditions: **9a** (0.5 mol%), KOH (10 mol%) in isopropanol at reflux [23]. Polysubstituted aryl aldehydes and aliphatic aldehydes could nevertheless be transfer hydrogenated by **9a** to the corresponding primary alcohols, but with much lower efficiency (TOF = 39–72 h^−1^; TON = 78–144) than what was observed with **11c** and **12** for benzaldehyde.

**Scheme 33:**
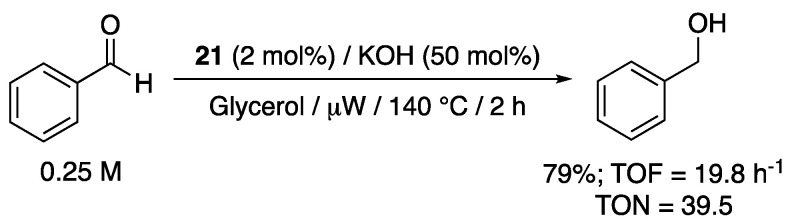
TH of benzaldehyde from glycerol catalyzed by the bis-sulfonated complex **21**.

The pyridyl-functionalized imidazol-2-ylidene complex **21**, bearing two propylsulfonate groups, that was shown by Voutchkova-Kostal et al. to be moderately active for the TH of acetophenone from glycerol under microwave heating at 140 °C (TOF = 7.5 h^−1^, TON = 15, Scheme 13), proved to be also active for the TH of benzaldehyde [32]. Thus, under the same reaction conditions, 79% yield to benzylic alcohol was achieved after 2 h reaction with 2 mol% **21** and 50 mol% KOH, giving TOF and TON values that are about 2.5 times higher than those obtained for the TH of acetophenone (Scheme 33). No aldol condensation product was reported.

## 5. Transfer Hydrogenation of Imines

Despite the importance of the resulting amines in the field of natural products, pharmaceuticals and agronomical compounds, the TH imines has rarely been studied with donor-functionalized NHC-ruthenium complexes, and has been limited to that of aldimines.

### 5.1. Aldimines TH with (κ^2^-C_NHC_,N)-Complexes

**Scheme 34:**
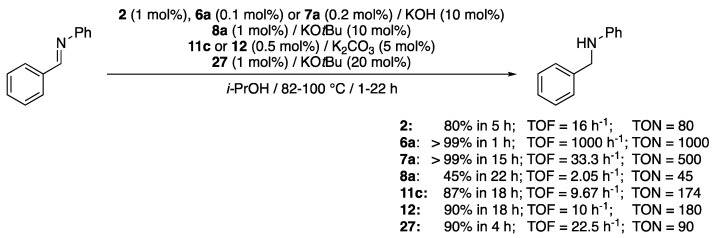
TH of *N*-benzylidineaniline catalyzed by complexes **2**, **6a**, **7a**, **8a**, **11c**, **12** and **27**.

To our knowledge, the first example with (*κ*^2^-*C_NHC_*,*N*)-complexes only appeared in 2009 with the pyrimidine-functionalized Ru(II)-NHC complex **2** (Scheme 2) disclosed by Crabtree et al. [12]. It was shown to reduce *N*-benzylideneaniline in 80% yield after 5 h reaction in isopropanol at reflux with a 1 mol% catalyst loading in the presence of 10 mol% KOH (Scheme 34). A couple of years later, Valerga and Puerta’s picolyl-imidazol-2-ylidene complexes **6a** and **7a** demonstrated a better efficacy, in particular the Cp* derivative **6a**, as it could fully reduce *N*-benzylideneaniline in 1 h with a catalyst loading of only 0.1 mol% under otherwise similar conditions, achieving a TOF of 1000 h^−1^ and a TON of 1000 (Scheme 34) [16,18]. The latter is still today the most efficient (*κ*^2^-*C_NHC_*,*N*)-precatalyst, as later reports from Hou [22] with his imino-imidazol-2-ylidene complex **8a**, Sarkar [25] with his pyridine- or pyrimidine-mesoionic carbene complexes **11c** and **12**, and Rit [35] with his triazole-imidazol-2-ylidene complex **27** only gave low to moderate activities (Scheme 34).

**Scheme 35:**
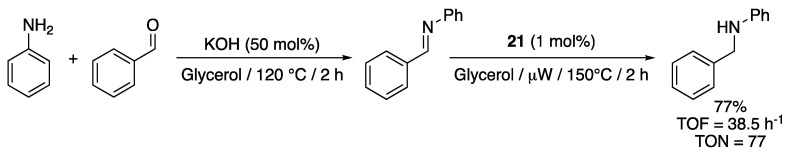
TH of *N*-benzylidineaniline from glycerol catalyzed by the bis-sulfonated complex **21**.

The versatile bis-sulfonated pyridyl-functionalized imidazole-2-ylidene complex **21** (1 mol%) catalyzed the TH of in situ prepared *N*-benzylideneaniline—using aniline and benzaldehyde—from glycerol under microwave irradiation at 150 °C giving *N*-benzylaniline in 77% yield after 2 h reaction (TOF = 38.5 h^−1^) (Scheme 35) [32]. To the best of our knowledge, this was the first report of TH of imines from glycerol. The reaction could also be carried out under conventional heating but only gave a poor 17% yield after 6 h reaction (TOF = 2.83 h^−1^). The important increase in activity brought by the microwave heating was tentatively attributed to an improved dispersion of the reactants and base in the polar and viscous glycerol solvent under these conditions, as well as to the highly polar zwitterionic character of **21**, which likely enhances microwave irradiation’s absorbance. Surprisingly, a control experiment carried out with a preformed imine in the absence of a base gave the reduced product in 63% yield under microwave irradiation. The authors suggested that this observed activity in the absence of KOH may result from the sulfonate arms acting as an internal base. No mechanistic investigation was however conducted to confirm this supposition.

### 5.2. Aldimines TH with (κ^2^-C_NHC_,O)-Complexes

**Scheme 36:**
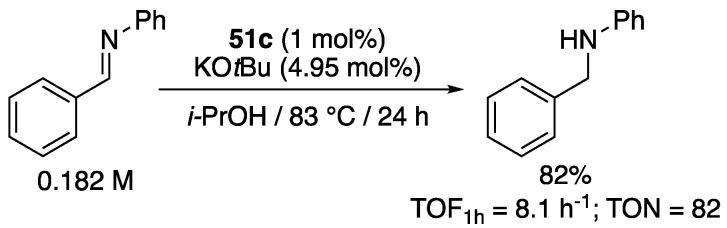
TH of *N*-benzylidineaniline catalyzed by the carboxylate-functionalized benzimidazol-2-ylidene complex **51c**.

As seen in Section 2.3 and Section 3.2, (*κ*^2^-*C_NHC_*,*O*)-complexes are rare, and we are aware of only one example of TH of imines with such complexes, that was described with the carboxylate-functionalized benzimidazol-2-ylidene complex **51c** (Figure 6) [68]. In the presence of 4.95 mol% KO*t*Bu, the latter proved able, with a loading of 1 mol%, and an initial TOF_1h_ after 1 h of 8.1 h^−1^, to reduce *N*-benzylidineaniline in 82% yield after 24 h reaction in isopropanol at reflux (Scheme 36), which is significantly slower and lower than what was observed with acetophenone (TOF_1h_ = 31 h^−1^, TON = 96) under similar conditions. However, this reduced activity with aldimines is in line with what was observed with the majority of the *κ*^2^-*C_NHC_*,*N*-complexes that proved able to catalyze the TH of imines, i.e., complexes **2**, **8a**, **11c**, and **12** (see Section 5.1).

## 6. Transfer Hydrogenation of Alkenes

Examples of ruthenium-catalyzed TH of C–C multiple bonds are exceedingly rare [7], and we are aware of only three examples with donor-functionalized *N*-heterocyclic carbene complexes, two that deal with the reduction in a unfunctionalized alkenes [25,72], and another one that deals with the TH of a polarized C=C double bond [68].

### 6.1. Alkenes TH with (κ^2^-C,N)-Complexes

**Scheme 37:**
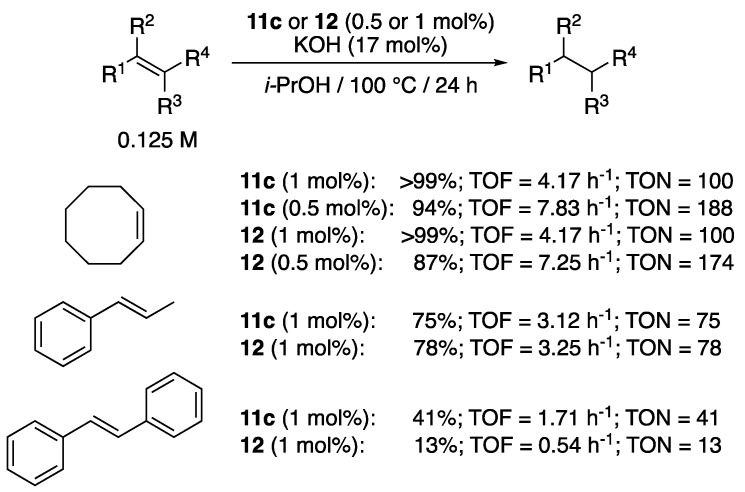
TH of alkenes by the pyridyl- and pyrimidyl-functionalized triazolylidene complexes **11c** and **12**.

The pyridyl- and pyrimidyl-functionalized mesoionic triazolylidene complexes **11c** and **12** proved also active for the TH of a couple of alkenes [25]. Notably, cyclooctene could be fully reduced to cyclooctane with either **11c** or **12** (1 mol%) in isopropanol at 100 °C for 24 h (TOF = 4.17 h^−1^) in the presence of a large amount of KOH (17 mol%) (Scheme 37). Excellent conversions were also observed with catalytic loadings of 0.5 mol%, in particular with the methoxy-functionalized pyridyl derivative **11c** (TOF = 7.83 h^−1^ and TON = 188). Lower efficiencies were however observed with both pre-catalysts for the reductions of *trans*-β-methylstyrene and *trans*-stilbene, with maximum TON values of 78 and 41, respectively.

### 6.2. Alkenes TH with (κ^2^-C,O)-Complexes

**Scheme 38:**
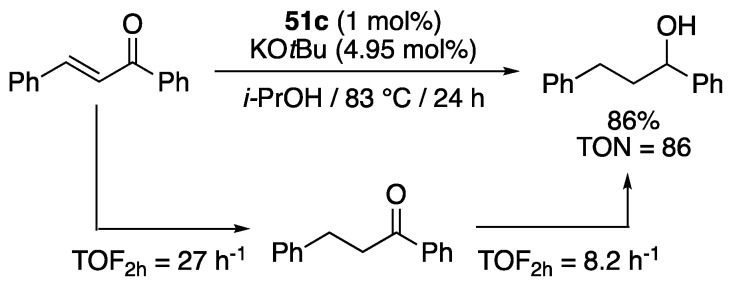
TH of chalcone catalyzed by the carboxylate-functionalized benzimidazol-2-ylidene complex **51c**.

The carboxylate-functionalized benzimidazol-2-ylidene complex **51c** (1 mol%) fully reduced the α,β-unsaturated ketone, chalcone, to 1,3-diphenyl-propan-1-ol in 86% yield in 24 h in isopropanol at reflux in the presence of KO*t*Bu (4.95 mol%) (Scheme 38). ^1^H NMR studies revealed that hydrogenation initially occurred at the C=C double bond and that 1,3-diphenylpropan-1-one was formed as the sole product with a TOF of 27 h^−1^ for the first 2 h. After 2h, 1,3-diphenyl-propan-1-ol began to appear from hydrogenation of the C=O bond of 1,3-diphenylpropan-1-one with a TOF of 8.2 h^−1^ for the next 2 h. Despite this encouraging result for the TH of alkenes, **51c** proved unable to reduce stilbene under similar conditions, which suggested that polarization of the C=C double bond was necessary to observe reactivity [68].

### 6.3. Alkenes TH with (κ^2^-C,S)-Complexes

**Scheme 39:**
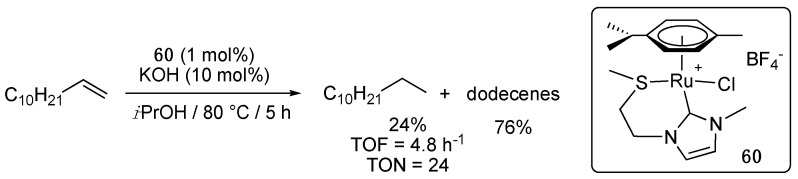
TH of 1-dodecene catalyzed by the thioether-functionalized imidazol-2-ylidene complex **60**.

The carboxylate-functionalized imidazole-2-ylidene complex **51a** and the thioether-functionalized imidazole-2-ylidene complex **60**, which were both prepared via transmetalation from the in situ generated adequate silver carbene, were evaluated as catalyst precursors (1 mol%) for the TH of the non-activated alkene, 1-dodecene, under classical TH conditions, i.e., in isopropanol as hydrogen source and solvent at 80 °C in the presence of KOH (10 mol%) [72]. Whereas **51a** was completely inactive under these conditions, **60** fully converted 1-dodecene to a mixture of dodecane (24%) and various dodecenes (76%) in 5 h (Scheme 39). However, prolonged heating to 24 h only allowed a slight increase in the hydrogenated product to 32%.

## 7. Miscellaneous Transfer Hydrogenation

**Scheme 40:**
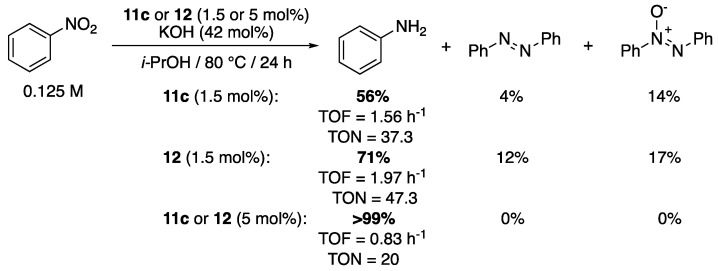
TH of nitrobenzene to aniline catalyzed by the pyridyl- and pyrimidyl-functionalized triazolylidene complexes **11c** and **12**.

The versatile pyridyl- and pyrimidyl-functionalized mesoionic triazolylidene complexes **11c** and **12** were also shown to be efficient precatalysts for the reduction in nitrobenzene to aniline [25]. In isopropanol at 80 °C, in the presence of 0.42 equiv. of KOH, the pyrimidyl derivative **12** (1.5 mol%), in particular, allowed the full conversion of nitrobenzene to aniline (71%), azobenzene (12%), and azoxybenzene (17%) in 24 h reaction, while **11c** proved slightly less reactive with only 56% conversion to aniline and, respectively, 4% and 14% to azobenzene and azoxybenzene (Scheme 40). When 5 mol% of either precatalyst was used, a dramatic improvement to full conversion of nitrobenzene to aniline was observed.

**Scheme 41:**
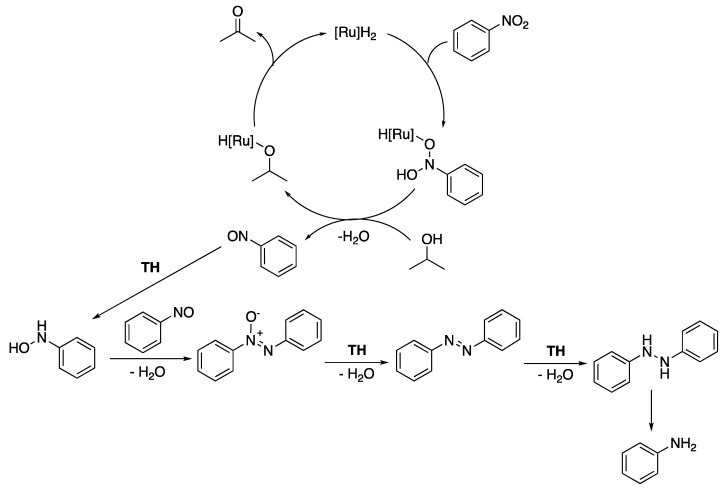
Proposed mechanism for the TH of nitrobenzene to aniline catalyzed by **11**c and **12**.

Based on a previously reported mechanistic study conducted with related arene-Ru(II) and -Ir(III) complexes bearing pyridyl-triazole or bis-triazole ligands [73], the authors proposed the mechanism depicted in Scheme 41. In this mechanism, the precatalysts would be first converted to ruthenium hydrides “[Ru]H_2_” in basic isopropanol. Following the formation of nitrosobenzene through a first TH, the latter would then be successively transfer hydrogenated to azoxybenzene, azobenzene, and aniline.

## 8. Conclusions

Bidentate donor-functionalized NHC-ruthenium complexes have been successfully used as precatalysts for the TH of ketones, aldehydes, aldimines, alkenes, and nitrobenzene. The most widely studied reaction by far is the TH of ketones, and a large number of (*κ*^2^-*C_NHC_*,*N*)-ruthenium precatalysts have been investigated for this transformation, comprising amine, pyridine-, pycolyl-, pyrimidine-, imine-, oxazoline-, pyrazole, triazole-, and pyridylideneamide-functionalized NHC ligands. (*κ*^2^-*C_NHC_*,*P*)- and (*κ*^2^-*C_NHC_*,*O*)-complexes as ketone TH precatalysts are, in contrast, relatively rare, and (*κ*^2^-*C_NHC_*,*S*)-complexes have curiously not been investigated. Nevertheless, the (diphenylphosphino)methylene-functionalized bis-imidazol-4-ylidene ruthenium complex **49** is by far the most active donor-functionalized NHC-ruthenium complex giving TOF as high as 198,000 h^−1^. The TH of aldehydes, aldimines, alkenes, and nitrobenzene, have also been mainly studied with the (*κ*^2^-*C_NHC_*,*N*)-ruthenium species. It thus appears from this review that, despite the easy modulation of NHCs, donor-functionalized NHC ligands comprising nitrogen atoms have attracted most of the attention, while the other donors have been clearly under-exploited, leaving a broad avenue for further improvements, in particular for the enantioselective variants of these reactions, which have been barely studied.

## References

[1] Wang D., Astruc D. (2015). The Golden Age of Transfer Hydrogenation. Chem. Rev..

[2] Zassinovich G., Mestroni G., Gladiali S. (1992). Asymmetric hydrogen transfer reactions promoted by homogeneous transition metal catalysts. Chem. Rev..

[3] Noyori R., Hashiguchi S. (1997). Asymmetric Transfer Hydrogenation Catalyzed by Chiral Ruthenium Complexes. Acc. Chem. Res..

[4] Clapham S.E., Hadzovic A., Morris R.H. (2004). Mechanisms of the H2-hydrogenation and transfer hydrogenation of polar bonds catalyzed by ruthenium hydride complexes. Coord. Chem. Rev..

[5] Gladiali S., Alberico E. (2005). Asymmetric transfer hydrogenation: Chiral ligands and applications. Chem. Soc. Rev..

[6] Hey D.A., Reich R.M., Baratta W., Kühn F.E. (2018). Current advances on ruthenium(II) N-heterocyclic carbenes in hydrogenation reactions. Coord. Chem. Rev..

[7] Ritleng V., de Vries J. (2021). Ruthenacycles and Iridacycles as Transfer Hydrogenation Catalysts. Molecules.

[8] Chernyshev V.M., Denisova E., Eremin D.B., Ananikov V.P. (2020). The key role of R–NHC coupling (R = C, H, heteroatom) and M–NHC bond cleavage in the evolution of M/NHC complexes and formation of catalytically active species. Chem. Sci..

[9] Normand A.T., Cavell K.J. (2008). Donor-Functionalised N-Heterocyclic Carbene Complexes of Group 9 and 10 Metals in Catalysis: Trends and Directions. Eur. J. Inorg. Chem..

[10] Neshat A., Mastrorilli P., Mobarakeh A.M. (2021). Recent Advances in Catalysis Involving Bidentate N-Heterocyclic Carbene Ligands. Molecules.

[11] Poyatos M., Maisse-François A., Bellemin-Laponnaz S., Peris E., Gade L.H. (2006). Synthesis and structural chemistry of arene-ruthenium half-sandwich complexes bearing an oxazolinyl–carbene ligand. J. Organomet. Chem..

[12] Gnanamgari D., Sauer E.L.O., Schley N., Butler C., Incarvito C.D., Crabtree R.H. (2008). Iridium and Ruthenium Complexes with Chelating N-Heterocyclic Carbenes: Efficient Catalysts for Transfer Hydrogenation, β-Alkylation of Alcohols, and N-Alkylation of Amines. Organometallics.

[13] Wylie W.N., Lough A.J., Morris R.H. (2009). Transmetalation of a Primary Amino-Functionalized N-Heterocyclic Carbene Ligand from an Axially Chiral Square-Planar Nickel(II) Complex to a Ruthenium(II) Precatalyst for the Transfer Hydrogenation of Ketones. Organometallics.

[14] Ohara H., Wylie W.N., Lough A.J., Morris R.H. (2012). Effect of chelating ring size in catalytic ketone hydrogenation: Facile synthesis of ruthenium(ii) precatalysts containing an N-heterocyclic carbene with a primary amine donor for ketone hydrogenation and a DFT study of mechanisms. Dalton Trans..

[15] Cross W.B., Daly C.G., Boutadla Y., Singh K. (2011). Variable coordination of amine functionalised N-heterocyclic carbene ligands to Ru, Rh and Ir: C–H and N–H activation and catalytic transfer hydrogenation. Dalton Trans..

[16] Fernández F.E., Puerta M.C., Valerga P. (2011). Half-Sandwich Ruthenium(II) Picolyl-NHC Complexes: Synthesis, Characterization, and Catalytic Activity in Transfer Hydrogenation Reactions. Organometallics.

[17] Ning J., Shang Z., Xu X. (2015). How Does the Hemilabile Group in Ruthenium-Cp* Picolyl-NHC Complexes Affect the Mechanism of Transfer Hydrogenation Reaction? A DFT Study. Catal. Lett..

[18] Fernández F.E., Puerta M.C., Valerga P. (2012). Ruthenium(II) Picolyl-NHC Complexes: Synthesis, Characterization, and Catalytic Activity in Amine N-alkylation and Transfer Hydrogenation Reactions. Organometallics.

[19] Piyasaengthong A., Williams L.J., Yufit D.S., Walton J.W. (2022). Novel ruthenium complexes bearing bipyridine-based and N-heterocyclic carbene-supported pyridine (NCN) ligands: The influence of ligands for catalytic transfer hydrogenation of ketones. Dalton Trans..

[20] Pámies O., Bäckvall J.-E. (2001). Studies on the Mechanism of Metal-Catalyzed Hydrogen Transfer from Alcohols to Ketones. Chem. Eur. J..

[21] Albrecht M., Miecznikowski J., Samuel A., Faller J., Crabtree R.H. (2002). Chelated Iridium(III) Bis-carbene Complexes as Air-Stable Catalysts for Transfer Hydrogenation. Organometallics.

[22] Guo X.-Q., Wang Y.-N., Wang D., Cai L.-H., Chen Z.-X., Hou X.-F. (2012). Palladium, iridium and ruthenium complexes with acyclic imino-N-heterocyclic carbenes and their application in aqua-phase Suzuki–Miyaura cross-coupling reaction and transfer hydrogenation. Dalton Trans..

[23] Delgado-Rebollo M., Canseco-Gonzalez D., Hollering M., Mueller-Bunz H., Albrecht M. (2013). Synthesis and catalytic alcohol oxidation and ketone transfer hydrogenation activity of donor-functionalized mesoionic triazolylidene ruthenium(ii) complexes. Dalton Trans..

[24] Bolje A., Hohloch S., van der Meer M., Košmrlj J., Sarkar B. (2015). Ru^II^, Os^II^, and Ir^III^Complexes with Chelating Pyridyl-Mesoionic Carbene Ligands: Structural Characterization and Applications in Transfer Hydrogenation Catalysis. Chem.-A Eur. J..

[25] Bolje A., Hohloch S., Košmrlj J., Sarkar B. (2016). Ru^II^, Ir^III^ and Os^II^ mesoionic carbene complexes: Efficient catalysts for transfer hydrogenation of selected functionalities. Dalton Trans..

[26] Olguín J., Paz-Sandoval M. (2017). Synthesis and transfer hydrogenation catalysis of chelating triazolylidene ruthenium(II) complexes: Effect of the pendant arm, p-cymene, acetonitrile and butadienesulfonyl co-ligands. J. Organomet. Chem..

[27] Sabater S., Müller-Bunz H., Albrecht M. (2016). Carboxylate-Functionalized Mesoionic Carbene Precursors: Decarboxylation, Ruthenium Bonding, and Catalytic Activity in Hydrogen Transfer Reactions. Organometallics.

[28] Olguín J., Díaz-Fernández M., de la Cruz-Cruz J.I., Paz-Sandoval M.A. (2016). Mixed heteropentadienyl and N-heterocyclic carbene ruthenium(II) complexes: Synthesis and transfer hydrogenation catalysis. J. Organomet. Chem..

[29] Smith I.G., Zgrabik J.C., Gutauskas A.C., Gray D.L., Domski G.J. (2017). Synthesis, characterization, and catalytic behavior of mono- and bimetallic ruthenium(II) and iridium(III) complexes supported by pyridine-functionalized N -heterocyclic carbene ligands. Inorg. Chem. Commun..

[30] Viji M., Tyagi N., Naithani N., Ramaiah D. (2017). Aryl appended neutral and cationic half-sandwich ruthenium(ii)–NHC complexes: Synthesis, characterisation and catalytic applications. New J. Chem..

[31] Castañón E.B., Kaposi M., Reich R.M., Kühn F.E. (2018). Water-soluble transition metal complexes of ruthenium(ii), osmium(ii), rhodium(iii) and iridium(iii) with chelating N-heterocyclic carbene ligands in hydrogenation and transfer hydrogenation catalysis. Dalton Trans..

[32] Azua A., Finn M., Yi H., Dantas A.B., Voutchkova-Kostal A. (2017). Transfer Hydrogenation from Glycerol: Activity and Recyclability of Iridium and Ruthenium Sulfonate-Functionalized N-Heterocyclic Carbene Catalysts. ACS Sustain. Chem. Eng..

[33] Nair A.G., McBurney R.T., Walker D.B., Page M.J., Gatus M.R.D., Bhadbhade M., Messerle B.A. (2016). Ruthenium(ii) complexes of hemilabile pincer ligands: Synthesis and catalysing the transfer hydrogenation of ketones. Dalton Trans..

[34] Hollering M., Albrecht M., Kühn F.E. (2016). Bonding and Catalytic Application of Ruthenium N-Heterocyclic Carbene Complexes Featuring Triazole, Triazolylidene, and Imidazolylidene Ligands. Organometallics.

[35] Illam P.M., Donthireddy S.N.R., Chakrabartty S., Rit A. (2019). Heteroditopic Ru(II)– and Ir(III)–NHC Complexes with Pendant 1,2,3-Triazole/Triazolylidene Groups: Stereoelectronic Impact on Transfer Hydrogenation of Unsaturated Compounds. Organometallics.

[36] Sinha A., Daw P., Rahaman S.W., Saha B., Bera J.K. (2011). A RuII–N-heterocyclic carbene (NHC) complex from metal–metal singly bonded diruthenium(I) precursor: Synthesis, structure and catalytic evaluation. J. Organomet. Chem..

[37] Yoshimura M., Kamisue R., Sakaguchi S. (2013). Synthesis of Ru(II) complexes containing N-heterocyclic carbenes functionalized with secondary donor groups: Catalytic activity toward enantioselective transfer hydrogenation. J. Organomet. Chem..

[38] Sortais J.-B., Ritleng V., Voelklin A., Holuigue A., Smail H., Barloy L., Sirlin C., Verzijl G.K.M., Boogers J.A.F., de Vries A.H.M. (2005). Cycloruthenated Primary and Secondary Amines as Efficient Catalyst Precursors for Asymmetric Transfer Hydrogenation. Org. Lett..

[39] Jerphagnon T., Haak R., Berthiol F., Gayet A.J.A., Ritleng V., Holuigue A., Pannetier N., Pfeffer M., Voelklin A., Lefort L. (2010). Ruthenacycles and Iridacycles as Catalysts for Asymmetric Transfer Hydrogenation and Racemisation. Top. Catal..

[40] Kilic A., Kayan C., Aydemir M., Durap F., Durgun M., Baysal A., Tas E., Gümgüm B. (2011). Synthesis of new boron complexes: Application to transfer hydrogenation of acetophenone derivatives. Appl. Organomet. Chem..

[41] Kilic A., Aydemir M., Durgun M., Meriç N., Ocak Y.S., Keles A., Temel H. (2014). Fluorine/phenyl chelated boron complexes: Synthesis, fluorescence properties and catalyst for transfer hydrogenation of aromatic ketones. J. Fluor. Chem..

[42] Leigh V., Carleton D.J., Olguin J., Mueller-Bunz H., Wright L.J., Albrecht M. (2014). Solvent-Dependent Switch of Ligand Donor Ability and Catalytic Activity of Ruthenium(II) Complexes Containing Pyridinylidene Amide (PYA) N-Heterocyclic Carbene Hybrid Ligands. Inorg. Chem..

[43] Donnelly K.F., Segarra C., Shao L.-X., Suen R., Müller-Bunz H., Albrecht M. (2015). Adaptive N-Mesoionic Ligands Anchored to a Triazolylidene for Ruthenium-Mediated (De)Hydrogenation Catalysis. Organometallics.

[44] Navarro M., Segarra C., Pfister T., Albrecht M. (2020). Structural, Electronic, and Catalytic Modulation of Chelating Pyridylideneamide Ruthenium(II) Complexes. Organometallics.

[45] Malan F.P., Singleton E., van Rooyen P.H., Albrecht M., Landman M. (2019). Synthesis, Stability, and (De)hydrogenation Catalysis by Normal and Abnormal Alkene- and Picolyl-Tethered NHC Ruthenium Complexes. Organometallics.

[46] Albrecht M. (2008). C4-bound imidazolylidenes: From curiosities to high-impact carbene ligands. Chem. Commun..

[47] Crabtree R.H. (2013). Abnormal, mesoionic and remote N-heterocyclic carbene complexes. Coord. Chem. Rev..

[48] Juzgado A., Lorenzo-García M.M., Barrejón M., Rodríguez A.M., Rodríguez-López J., Merino S., Tejeda J. (2014). Chelation assistance as a tool for the selective preparation of an imidazole-based mesoionic palladium carbene complex. Chem. Commun..

[49] Cheng Y., Sun J.-F., Yang H.-L., Xu H.-J., Li Y.-Z., Chen X.-T., Xue Z.-L. (2009). Syntheses, Structures, and Catalytic Properties of Ruthenium(II) Nitrosyl Complexes with Pyridine-Functionalized N-Heterocyclic Carbenes. Organometallics.

[50] Cheng Y., Xu H.-J., Sun J.-F., Li Y.-Z., Chen X.-T., Xue Z.-L. (2009). Synthesis, structures and catalytic activities of ruthenium(ii) carbonyl chloride complexes containing pyridine-functionalised N-heterocyclic carbenes. Dalton Trans..

[51] Li X.-W., Wang G.-F., Chen F., Li Y.-Z., Chen X.-T., Xue Z.-L. (2011). Ruthenium(II) carbonyl chloride complexes containing pyridine-functionalised bidentate N-heterocyclic carbenes: Synthesis, structures, and impact of the carbene ligands on catalytic activities. Inorg. Chim. Acta.

[52] Chen C., Lu C., Zheng Q., Ni S., Zhang M., Chen W. (2015). Synthesis and structures of ruthenium–NHC complexes and their catalysis in hydrogen transfer reaction. Beilstein J. Org. Chem..

[53] Miranda-Soto V., Grotjahn D.B., Cooksy A.L., Golen J.A., Moore C.E., Rheingold A.L. (2010). A Labile and Catalytically Active Imidazol-2-yl Fragment System. Angew. Chem. Int. Ed..

[54] Kuwata S., Ikariya T. (2014). Metal-ligand bifunctional reactivity and catalysis of protic N-heterocyclic carbene and pyra-zole complexes featuring β-NH units. Chem. Commun..

[55] Humphries M.E., Pecak W.H., Hohenboken S.A., Alvarado S.R., Swenson D.C., Domski G.J. (2013). Ruthenium(II) supported by phosphine-functionalized N-heterocyclic carbene ligands as catalysts for the transfer hydrogenation of ketones. Inorg. Chem. Commun..

[56] Witt J., Pöthig A., Kühn F.E., Baratta W. (2013). Abnormal N-Heterocyclic Carbene-Phosphine Ruthenium(II) Complexes as Active Catalysts for Transfer Hydrogenation. Organometallics.

[57] Yamakawa M., Ito H., Noyori R. (2000). The Metal−Ligand Bifunctional Catalysis: A Theoretical Study on the Ruthenium(II)-Catalyzed Hydrogen Transfer between Alcohols and Carbonyl Compounds. J. Am. Chem. Soc..

[58] Bitzer M.J., Pöthig A., Jandl C., Kühn F.E., Baratta W. (2015). Ru–Ag and Ru–Au dicarbene complexes from an abnormal carbene ruthenium system. Dalton Trans..

[59] Pardatscher L., Bitzer M.J., Jandl C., Kück J.W., Reich R.M., Kühn F.E., Baratta W. (2018). Cationic abnormal N-heterocyclic carbene ruthenium complexes as suitable precursors for the synthesis of heterobimetallic compounds. Dalton Trans..

[60] Pardatscher L., Hofmann B.J., Fischer P.J., Hölzl S.M., Reich R.M., Kühn F.E., Baratta W. (2019). Highly Efficient Abnormal NHC Ruthenium Catalyst for Oppenauer-Type Oxidation and Transfer Hydrogenation Reactions. ACS Catal..

[61] Chelucci G., Baldino S., Baratta W. (2015). Ruthenium and osmium complexes containing 2-(aminomethyl)pyridine (Ampy)-based ligands in catalysis. Coord. Chem. Rev..

[62] Baratta W., Schütz J., Herdtweck E., Herrmann W.A., Rigo P. (2005). Fast transfer hydrogenation using a highly active orthometalated heterocyclic carbene ruthenium catalyst. J. Organomet. Chem..

[63] Poyatos M., Mata J.A., Falomir E., Crabtree R.H., Peris E. (2003). New Ruthenium(II) CNC-Pincer Bis(carbene) Complexes: Synthesis and Catalytic Activity. Organometallics.

[64] Hollering M., Weiss D.T., Bitzer M.J., Jandl C., Kühn F.E. (2016). Controlling Coordination Geometries: Ru–Carbene Complexes with Tetra-NHC Ligands. Inorg. Chem..

[65] Horn S., Gandolfi C., Albrecht M. (2011). Transfer Hydrogenation of Ketones and Activated Olefins Using Chelating NHC Ruthenium Complexes. Eur. J. Inorg. Chem..

[66] Gandolfi C., Heckenroth M., Neels A., Laurenczy G., Albrecht M. (2009). Chelating NHC Ruthenium(II) Complexes as Robust Homogeneous Hydrogenation Catalysts. Organometallics.

[67] DePasquale J., White N.J., Ennis E.J., Zeller M., Foley J.P., Papish E.T. (2012). Synthesis of chiral N-heterocyclic carbene (NHC) ligand precursors and formation of ruthenium(II) complexes for transfer hydrogenation catalysts. Polyhedron.

[68] Mangalum A., McMillen C.D., Tennyson A. (2015). Synthesis, coordination chemistry and reactivity of transition metal complexes supported by a chelating benzimidazolylidene carboxylate ligand. Inorg. Chim. Acta.

[69] Akkoç M., Öz E., Demirel S., Dorcet V., Roisnel T., Bayri A., Bruneau C., Altin S., Yaşar S., Özdemir I. (2018). Investigation of potential hybrid capacitor property of chelated N-Heterocyclic carbene Ruthenium(II) complex. J. Organomet. Chem..

[70] Pretorius R., Mazloomi Z., Albrecht M. (2017). Synthesis, hemilability, and catalytic transfer hydrogenation activity of iridium(III) and ruthenium(II) complexes containing oxygen-functionalised triazolylidene ligands. J. Organomet. Chem..

[71] Wan K.Y., Lough A.J., Morris R.H. (2016). Transition Metal Complexes of an (S,S)-1,2-Diphenylethylamine-Functionalized N-Heterocyclic Carbene: A New Member of the Asymmetric NHC Ligand Family. Organometallics.

[72] Horn S., Albrecht M. (2011). Transfer hydrogenation of unfunctionalised alkenes using N-heterocyclic carbene ruthenium catalyst precursors. Chem. Commun..

[73] Hohloch S., Suntrup L., Sarkar B. (2013). Arene–Ruthenium(II) and −Iridium(III) Complexes with “Click”-Based Pyridyl-triazoles, Bis-triazoles, and Chelating Abnormal Carbenes: Applications in Catalytic Transfer Hydrogenation of Nitrobenzene. Organometallics.

